# Laminarin is a major molecule in the marine carbon cycle

**DOI:** 10.1073/pnas.1917001117

**Published:** 2020-03-13

**Authors:** Stefan Becker, Jan Tebben, Sarah Coffinet, Karen Wiltshire, Morten Hvitfeldt Iversen, Tilmann Harder, Kai-Uwe Hinrichs, Jan-Hendrik Hehemann

**Affiliations:** ^a^MARUM Center for Marine Environmental Sciences, University of Bremen, 28359 Bremen, Germany;; ^b^Max Planck Institute for Marine Microbiology, 28359 Bremen, Germany;; ^c^Department of Geosciences, University of Bremen, 28359 Bremen, Germany;; ^d^Alfred Wegener Institute, Helmholtz Centre for Polar and Marine Research, 27570 Bremerhaven, Germany;; ^e^Faculty of Biology and Chemistry, University of Bremen, 28359 Bremen, Germany

**Keywords:** carbon cycle, laminarin, diatoms, glycans, diel cycle

## Abstract

Marine microalgae sequester as much CO_2_ into carbohydrates as terrestrial plants. Polymeric carbohydrates (i.e., glycans) provide carbon for heterotrophic organisms and constitute a carbon sink in the global oceans. The quantitative contributions of different algal glycans to cycling and sequestration of carbon remain unknown, partly because of the analytical challenge to quantify glycans in complex biological matrices. Here, we quantified a glycan structural type using a recently developed biocatalytic strategy, which involves laminarinase enzymes that specifically cleave the algal glycan laminarin into readily analyzable fragments. We measured laminarin along transects in the Arctic, Atlantic, and Pacific oceans and during three time series in the North Sea. These data revealed a median of 26 ± 17% laminarin within the particulate organic carbon pool. The observed correlation between chlorophyll and laminarin suggests an annual production of algal laminarin of 12 ± 8 gigatons: that is, approximately three times the annual atmospheric carbon dioxide increase by fossil fuel burning. Moreover, our data revealed that laminarin accounted for up to 50% of organic carbon in sinking diatom-containing particles, thus substantially contributing to carbon export from surface waters. Spatially and temporally variable laminarin concentrations in the sunlit ocean are driven by light availability. Collectively, these observations highlight the prominent ecological role and biogeochemical function of laminarin in oceanic carbon export and energy flow to higher trophic levels.

## Significance

Microscopic planktonic algae are the base of the marine food web. Although sugars are the most abundant biomolecules in land plants, their concentrations in marine plants appear surprisingly low. We used recently discovered enzymes to dissect microalgae inhabiting the sunlit ocean and found that 26 ± 17% of their biomass consists of the sugar polymer laminarin. The concentration in algal cells increased markedly during the day, in analogy to the seasonal storage of energy in starchy roots and fruits of land plants. Vast quantities of laminarin discovered in the ocean underscore the importance of marine sugars in the global carbon cycle. This work has implications for our understanding of the elemental stoichiometry of microalgae, the most important oceanic food source.

The production rate of organic carbon is controlled by the growth of photosynthetic microalgae in the sunlit ocean, where diatoms alone contribute about 40% of the marine primary production and convert equal amounts of carbon dioxide into biomass as tropical forests (1). Glycans are carbohydrates composed of multiple, linked monosaccharides, such as the glucose polysaccharide laminarin, which is a central energy metabolite in microalgae, including diatoms (2). Glycans are among the most abundant molecules synthesized by microalgae, yet the concentrations of glycan structural types have not yet been directly determined and thus quantitative oceanic glycan budgets remain poorly understood.

Bulk measurements by NMR and acid hydrolysis suggest algal glycans are crucial for carbon export and sequestration because they can aggregate into sinking particulate organic matter (POM) (3, 4). Glycans are also found dissolved in surface waters, indicating their potential to store carbon in high molecular weight dissolved organic matter (HMW-DOM) (4). Furthermore, they are important structural components, as well as carbon and energy metabolites, for algae and a source of nutrition for heterotrophs (5). However, we do not know which glycan structural types are the important food sources and aggregate into sinking particles, accumulate in HMW-DOM, or export carbon as part of sinking algal cells. Our understanding of glycans in POM and HMW-DOM remains limited because it is based largely on the characterization of monomers (6), and this information alone is insufficient to reconstruct glycan structural types and their abundance (7–9).

Existing estimates of quota of glycans in marine organic matter range between 10 to 75% of the total carbon depending on the sampling site, sampling strategy, and how the samples were analyzed (*SI Appendix*, Fig. S1). A widely used method for the quantification of glycans in marine organic matter uses acid hydrolysis, followed by colorimetric or liquid chromatographic determination of the constituent monosaccharides (10). Limitations of the acid hydrolysis method have been discussed since the 1960s, with the consensus being that it underestimates the carbohydrate concentration due to incomplete hydrolysis (11), destruction of the monosaccharides, and acid-catalyzed side reactions (12) between the sugar aldehyde and the amine groups of proteins. Considering glucose is the direct product of the photosynthesis reaction and thus the globally most synthesized biological building block, lower estimates of carbohydrate concentrations of about 10 to 15%, especially in regions of regular algae blooms, are of concern (13). New, less destructive, more efficient and specific bioanalytic tools may help to properly constrain the marine glyco-carbon cycle.

Heterotrophic bacteria have evolved to exploit the diversity of glycans by developing proteins adapted to accommodate the three-dimensional structure of their cognate glycan for binding (14) and catalysis (15). Taking advantage of this extraordinary specificity, we have recently developed a biocatalytic assay based on enzymes that selectively recognizes the glycan, such as laminarin, in marine organic matter (*SI Appendix*, Fig. S2) (16–18). Laminarin is a highly water soluble, branched polysaccharide made of a linear β-(1→3)–linked glucose-based chain with an average degree of polymerization (DP) of ∼20 to 30. We used endo-acting β-1,3-glucanases (glycoside hydrolase [GH] family 17), which specifically cleave β-(1→3) linkages. Laminarin also contains β-(1→6)–linked side chains consisting of one or more glucose moiety (*SI Appendix*, Fig. S2). These side chains are cleaved off by enzymes that are exo-acting β-1,6-glucosidases (GH30). A β-(1→3)–specific exo-glucosidase of family GH3 can further cleave the remaining oligosaccharides into glucose. The reaction products (i.e., glucose and remaining oligosaccharides) are readily measurable and proportional to the amount of laminarin in the sample (17, 18).

**Fig. 1.**
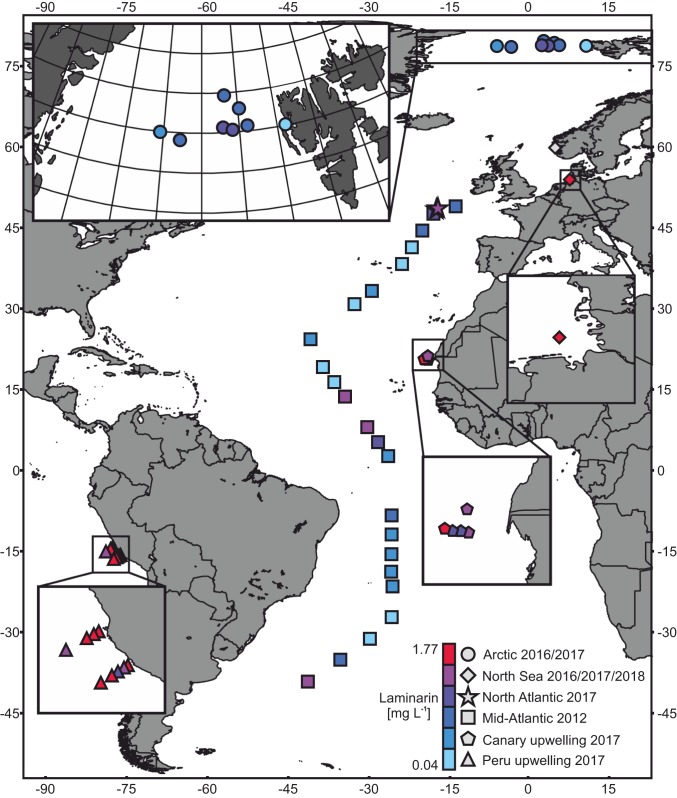
Station and laminarin surface concentration overview. The entire dataset comprised samples from nine different cruises and campaigns indicated by different symbols. The colors represent mean laminarin concentrations from surface samples (max. 50 m water depth). No average value was taken for the hourly sampling during the North Sea 2018 campaign in Norway. The enlarged Arctic region is mapped with a polar stereographic projection of the earth whereas the rest of the map and all other enlarged areas are shown in equirectangular projection. Black lines in the gray landmasses mark country borders. The map was created using QGIS (v.2.18.14) and the Natural Earth free vector and raster map.

We measured laminarin concentrations extracted from POM collected during six cruises to the Arctic in 2016 and 2017, North Atlantic, Peru upwelling, Canary upwelling, meridional Atlantic transect, in the Raunefjorden near Bergen, and during two time series in the North Sea near the island Helgoland, resulting in a total of over 250 samples from 51 stations (Fig. 1). In short, POM was collected in different size fractions via filtration and extracted with enzyme buffer followed by enzymatic hydrolysis and quantification. For example in the North Sea, POM was separated by sequential filtration into fractions larger than 10 µm, 10 to 3 µm, 3 to 0.2 µm, and additionally into one >0.7-µm fraction. Further, HMW-DOM samples were gained by subsequent concentration using a 1-kilodalton (kDa) membrane. In the sunlit ocean, microalgae account for most of the POM in the fraction larger than 0.7 µm. Smaller eukaryotic and prokaryotic species can be found in the <3-µm fraction (e.g., the green algae *Ostreococcus tauri*) whereas diatoms contribute mainly to the biomass in the >3-µm fraction (e.g., *Thalassiosira pseudonana*) and >10-µm fraction (e.g., *Coscinodiscus wailesii*). Submicrometer particles are mostly derived from nonliving organic matter (19). Sampling of small crustaceans (e.g., copepods) was prevented either by additional metal meshes or by visual examination of the filter. The majority of samples were taken in surface waters between 0 and 40 m depth (detailed sample information is summarized in Dataset S1). Only in the two Arctic and the North Atlantic datasets, samples were obtained from water depths of 350 m below the surface. The data for each of these cruises and the time series are presented in *SI Appendix*, Table S1 and Dataset S2. The laminarin quantification method was based on specific enzyme hydrolysis and previously developed and validated (17, 18). In the present study, it was further validated versus the classic acid hydrolysis method (*SI Appendix*, Fig. S3). A detailed description of these experiments and their results is found in the supplementary information (*SI Appendix*, Figs. S3–S6).

## Diatoms Drive Laminarin Production during Algal Spring Blooms in the North Sea.

**Fig. 2.**
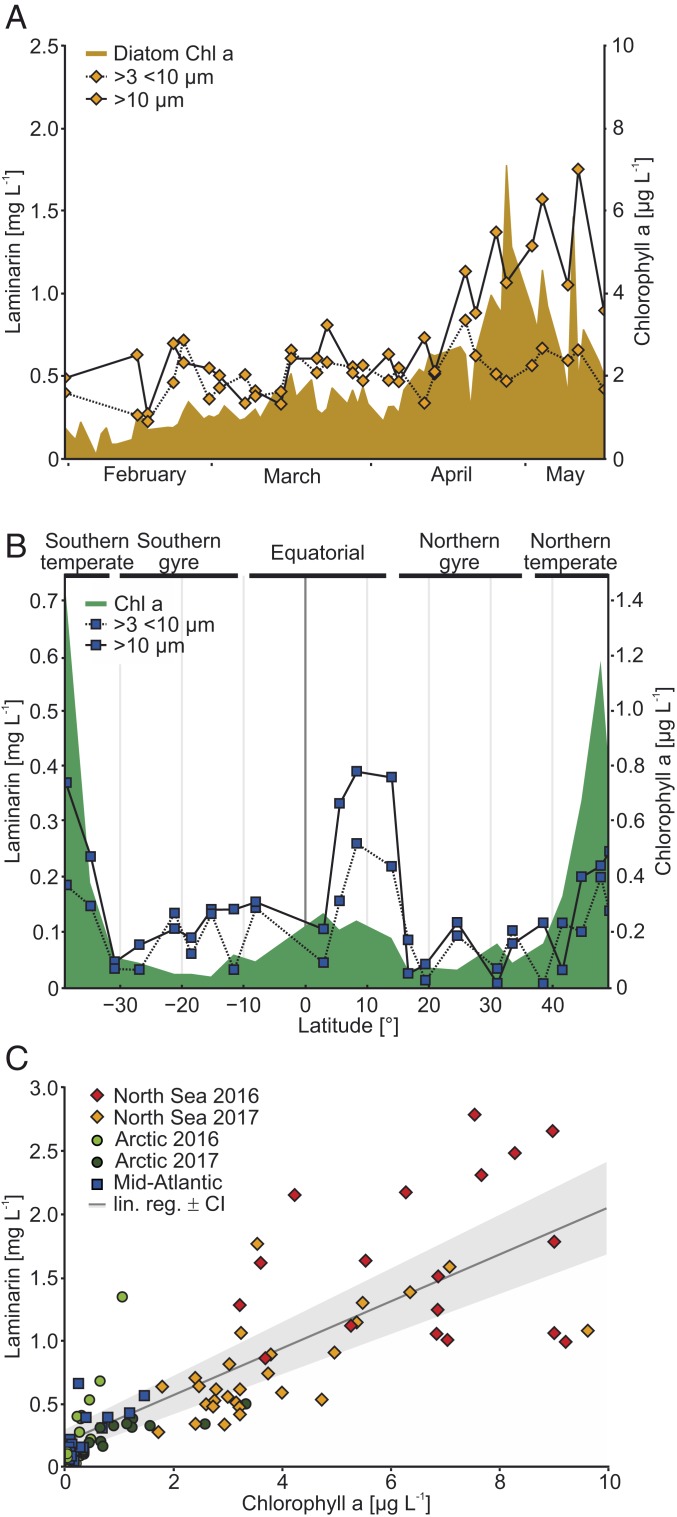
Laminarin and Chl-a concentrations correlate in a North Sea spring bloom time series, in the Atlantic, and in the Arctic. (*A*) Laminarin and Chl-a were determined in different size fractions during a phytoplankton spring bloom in the North Sea in 2017. (*B*) Laminarin was measured in different size fractions along a meridional transect from the North to the South Atlantic. (*C*) Comparison of laminarin and chlorophyll concentrations in all datasets where Chl-a was measured. Linear regression was applied to the laminarin-to-Chl-a relationship (*R*^2^ = 0.66; *P* < 0.001, *n* = 101). The confidence interval (CI) in gray was calculated at level 0.95.

We monitored laminarin in the North Sea by sampling during spring in 2016 and 2017 (Fig. 2). This time series showed some of the highest laminarin concentrations of the entire dataset (∼2.6 mg⋅L^−1^; overall mean 0.77 ± 0.66 mg⋅L^−1^), with laminarin present in microalgae size fractions between 3 and 10 µm, but not detectable in smaller size fractions. The laminarin increase during the 2017 bloom was driven by microalgae larger than 10 µm (linear regression: *R*^2^ = 0.40; *P* < 0.001, *n* = 28) (Fig. 2*A* and *SI Appendix*, Figs. S7*A* and S8*A*). This result was supported by multiwavelength spectrofluorometric analysis (20), which provided an estimate of the relative proportion of pigments derived from diatoms, chlorophytes, cryptophytes, and cyanobacteria (*SI Appendix*, Fig. S7*B*). Unlike diatoms, chlorophytes, which are known to primarily produce the polysaccharide starch (21), stayed at relatively constant levels (*SI Appendix*, Fig. S8*B*). Quantification of starch with an enzymatic assay based on amylase (EC 3.2.1.1) revealed that its concentrations were up to 10 times lower than those of laminarin, underscoring the importance of laminarin as a major glycan in the ocean (*SI Appendix*, Fig. S9).

## Laminarin Contributes Significantly to Particulate Organic Carbon in Surface Waters.

To further develop our understanding of the relationship between algal biomass and laminarin, we used combined chlorophyll A (Chl-a) as growth proxy (1) for algae (*SI Appendix*, Figs. S10 and S11) and found a linear correlation (*R*^2^ = 0.66; *P* < 0.001, *n* = 101) (Fig. 2*C*). We analyzed Arctic samples from 2016 and 2017 (*SI Appendix*, Fig. S10), as well as samples from a meridional Atlantic transect (Fig. 2*B*). Chl-a concentration ranged between ∼0.05 µg⋅L^−1^ in the two oligotrophic North and South Atlantic gyre regions and ∼1.4 µg⋅L^−1^ in temperate regions (Fig. 2*B*). Between the gyres in the northern equatorial upwelling region with higher productivity (22–24), the laminarin concentrations were elevated (∼0.37 on 10-µm and ∼0.21 mg⋅L^−1^ on 3-µm-size filters). Similar to the North Sea, the size fraction over 10 µm, which contains larger cells such as diatoms, contained more laminarin than the 3-µm-size fraction.

**Fig. 3.**
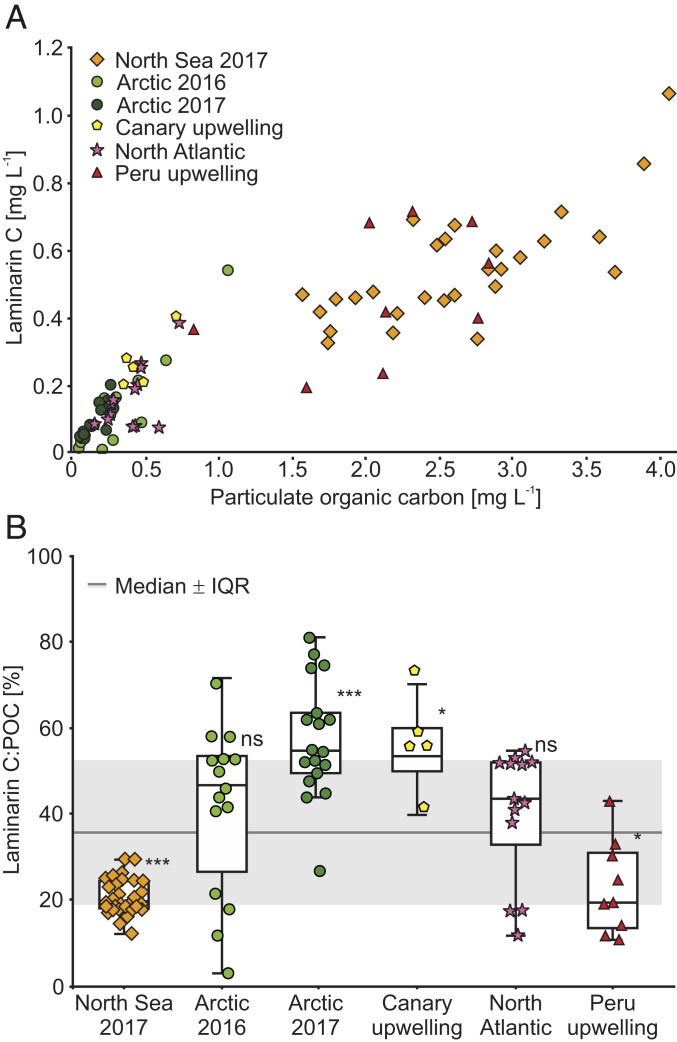
Laminarin is a substantial component of particulate organic carbon in diverse oceanic regions. (*A*) The overview scatter plot comprises all regions where POC was measured. (*B*) The box plot depicts each individual dataset against the overall median value of Laminarin C:POC. Significant deviations from the overall median and its standard deviation (SD) in gray were tested using the Kruskal–Wallis test (***P < 0.001; *P < 0.05; ns, not significant).

Laminarin-carbon (LamC) and particulate organic carbon (POC) are linearly correlated (*R*^2^ = 0.81; *P* < 0.001, *n* = 88) (Fig. 3*A* and *SI Appendix*, Figs. S12 and S13). However, LamC:POC ratios and the slopes of the linear correlations observed in the various settings (*SI Appendix*, Fig. S12) reveal variable laminarin concentrations relative to POC and, hence, that the bioenergy available to higher trophic species is temporally and regionally variable. Intriguingly, the datasets from the North Sea and Peru upwelling zone, both of which had among the highest concentrations of POC and laminarin, were characterized by the lowest LamC:POC ratios of 21 ± 6% (SD) and significantly deviated from the overall LamC:POC median of 37 ± 17% (interquartile range [IQR]) (*P* < 0.001 and *P* < 0.05, Kruskal–Wallis test; *n* = 88) (Fig. 3*B*). In these nutrient-rich regions, rapidly growing microalgae may reduce laminarin storage (25). The Arctic 2017 and the Canary upwelling sites showed LamC:POC ratios of around 58 ± 13% (SD), exceeding the median significantly (*P* < 0.001 and *P* < 0.05, Kruskal–Wallis test). Our data suggest a number of reasons, including nutrient concentrations and time of day when samples were taken as important controllers of the LamC:POC ratio.

## Diurnal Turnover of Laminarin in Phytoplankton.

**Fig. 4.**
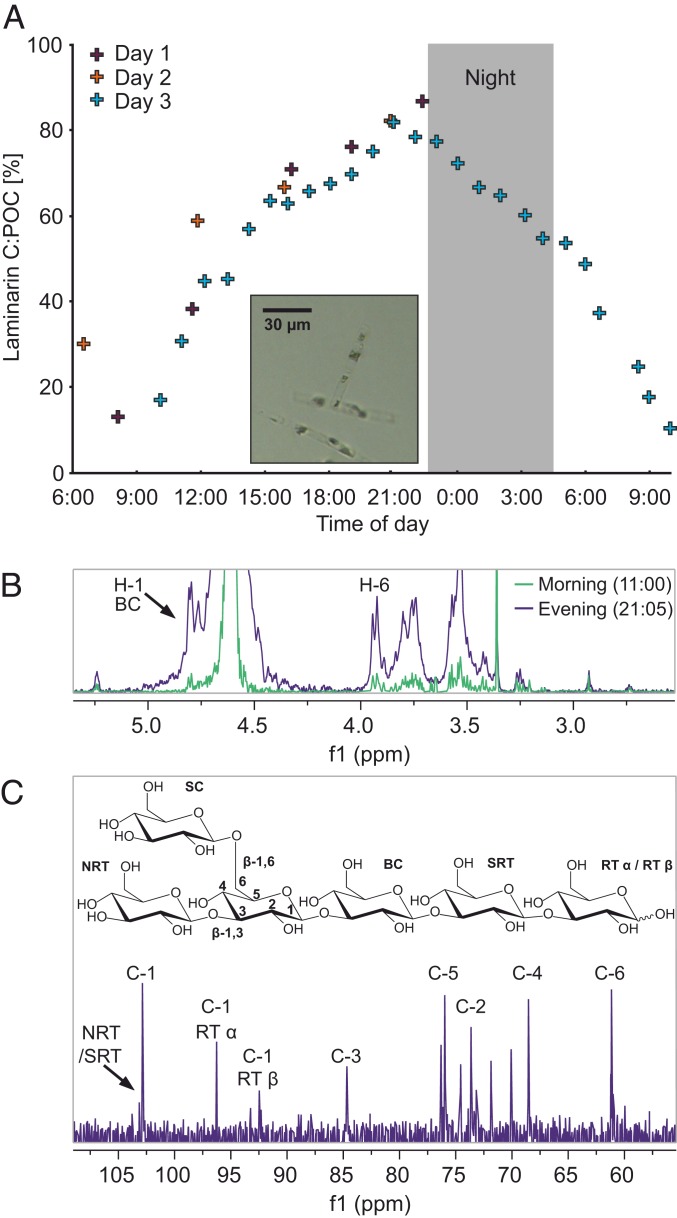
Diel-laminarin-cycling in the particulate organic carbon pool. (*A*) The scatter plot depicts the dataset of laminarin carbon per POC (LamC:POC) in % against the time of day. The gray area marks the time of sunrise and sunset. The sampling took place in spring 2018 during 3 d in the Raunefjorden near Bergen, Norway. On day 3, the sampling was conducted every hour for 24 h. The *Inset* shows a representative photograph of chain-forming diatoms that dominated the algal bloom. (*B*) ^1^H NMR spectra from two time points at 11:00 (green) and 21:05 (purple) showing the anomeric H-1 doublet of the backbone chain (BC) and H-6 (5 mg⋅mL^−1^ in D_2_O recorded at 600.2 MHz, 313 K, spectra normalized on the area of the ISTD). (*C*) ^13^C NMR spectrum of the 21:05 sample (250 mg⋅mL^−1^ in D_2_O recorded at 150.94 MHz, 313 K). POC filters were extracted with water at 60 °C. NRT(SRT), (second next to) nonreducing terminus.

The variation of LamC:POC may be explained by light availability. Light-driven increase of intracellular laminarin during the day and decrease during the night has been previously observed in laboratory cultures of diatoms (25–29). To explore the hypothesis that light was an important driver of laminarin concentration in phytoplankton POM, we conducted an experiment in which we measured LamC:POC in surface waters of the Raunefjorden (Norway, Bergen) during a spring diatom bloom (2018). We recorded the diel cycle of laminarin with hourly measurements for 24 h. POC analysis of the filters before and after extraction showed that 84% of the POC was soluble in MilliQ water at 21:05 PM and only 37% at ∼11:00 AM, which is consistent with the different laminarin concentrations at these time points. The LamC:POC ratio was ∼10% in the morning and ∼80% in the evening (Fig. 4*A*). These numbers are consistent with the range of values measured in other, highly productive regions like the North Sea during the spring bloom or upwelling regions (Fig. 3*B*).

NMR analyses confirmed the high proportion of LamC in water-soluble extracts of POM (Fig. 2*C*). Anomer resonances of laminarin at 4.29 to 4.98 parts per million (ppm) corresponded to literature values observed in cultivated diatoms (30). The resonance of the (1→3) backbone chain (BC) was at 4.54 ppm. The reducing α-anomer (RT) signal was at 4.93 ppm, and the β-anomer signal was at 4.42 ppm. The resonance from the terminal β-(1→6)–linked side chain groups (TSC) was at 4.29 ppm. We calculated a degree of polymerization (DP) of 15 and degree of branching (DB) of 0.1, which aligns with previously published values for laminarin from diatoms (31). NMR quantification via the H-6 proton of the backbone chain (BC) also confirmed the high differences in LamC between the morning (0.8 µmol⋅L^−1^) and the end of the day (7.7 µmol⋅L^−1^) (Fig. 4*B*). Notably, most proton (Fig. 4*B*) and carbon signals (Fig. 4*C*) could be assigned to the glucose monomers of laminarin (84% of the POC), consistent with the values from the enzyme assay. LamC is built up at a rate of ∼0.34 ± 0.03 mg⋅L^−1^⋅h^−1^ (SD) (for simplicity, rate assumed to be linear; equals ∼12.5 ± 0.1 nmol⋅L^−1^⋅h^−1^ [SD] as glucose equivalents) and afterward consumed at a rate of ∼0.35 mg⋅L^−1^⋅h^−1^. This diel variation of the LamC:POC ratio (Fig. 4*A*) indicates the high turnover of laminarin within phytoplankton OM. Thus, laminarin turnover within phytoplankton cells may account for at least some of the variability of LamC:POC ratios observed among the samples from the various oceanic regions (Fig. 3*B*). In conclusion, both the NMR experiment and the biocatalytic assay showed substantial diurnal fluctuation of laminarin within living diatom cells. Consequently, the time-resolved LamC:POC ratio may be a suitable indicator of carbon turnover in diatom-rich surface waters.

We further verified the quantitative contribution of laminarin to POC by mass balance of organic carbon in lipids, proteins, and laminarin. On a subset of 15 samples from the cruise to the Arctic in 2016, we determined protein and lipid masses and converted them into carbon equivalents (Table 1). In addition to the 42 ± 21% (SD) contribution from laminarin-carbon to the total POC pool, 44 ± 14% (SD) of the POC consisted of protein carbon and 4 ± 3% (SD) of lipid carbon. Together, laminarin, proteins and lipids accounted for about 90% total POC, leaving 10% of the POC to be composed of additional polysaccharides (e.g., cell wall), DNA, RNA, and other organic molecules.

**Table 1.** Proportions of lipid, protein, and laminarin and/or total carbohydrates in Arctic organic matter and microalgae derived POC (55, 80)

	Total carbohydrates, %	Laminarin, %	Proteins, %	Lipids, %	Uncharacterized, %
Parsons et al., 1961 (80)	23 ± 11	—	39 ± 13	8 ± 5	30 ± 29
Finkel et al., 2016 (55)	15 ± 11	—	32 ± 14	17 ± 10	36 ± 35
This study (Arctic 2016)	53 ± 16	***42 ± 21***	44 ± 14	4 ± 3	0 ± 33/***10 ± 33***

Shown are mean values and SDs. This analysis was only applied on the Arctic 2016 dataset (*n* = 15). Parsons et al. (80) and this study compared macromolecules with the amount of POC whereas the data in Finkel et al. (55) was compared to the total dry weight. Dashes illustrate that values are not available. Normal text indicates total carbohydrate contribution and based on that the respective approximation of the uncharacterized fraction. In contrast, italic bold numbers represent laminarin contribution and its respective uncharacterized fraction. Sums can exceed 100% due to truncation.

**Fig. 5.**
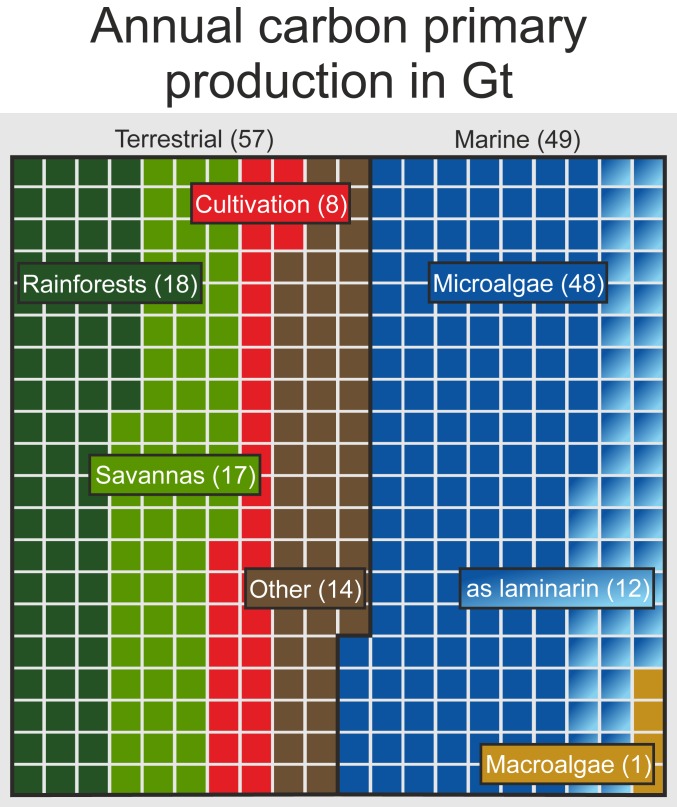
Net primary production (NPP) pools of carbon after Field et al. (1). Shown are major biological contributors from terrestrial and marine sources, normalized to 400 equal squares, each depicting ∼0.27 gigatons of carbon, which is annually being fixed by primary production; 12 ± 8% of the global carbon production is deposited in the form of the microalgal storage compound laminarin.

We further tested the validity of the laminarin quantification by checking their consistency with measured C/N values for a subset of samples (*SI Appendix*, Fig. S14). The elemental composition of particulate organic matter is constrained by the content of the major macromolecules glycans, proteins, lipids, and DNA leading to a mean Redfield ratio of 6.6 (32). Despite C/N values from 2.4 and 24, the mean C/N ratio of the dataset (8 ± 4) is close to the canonical Redfield ratio. Assuming that POM is a two-component mixture of the major pools glycan and protein, we can estimate a maximum permitted N content for any given laminarin concentration using the equation C/N = 3.82 × (1 − f_lam_), with 3.82 being the theoretical average C/N value of protein (33) and f_lam_ representing the fractional abundance of laminarin. This approach identified samples with possibly overestimated laminarin concentration. If we only consider the ∼70% of samples in which laminarin is consistent with the C/N ratio, we arrive at a median LamC:POC ratio of 26 ± 17% (mean 34 ± 17%). Assuming steady-state dynamics and using the median LamC:POC ratio of 26 ± 17% and the correlation of laminarin with Chl-a (Fig. 2*C*), we estimate that 12 ± 8 gigatons of the 47.5 gigatons of annual, marine primary production (1), or 11 ± 8% of the global primary production, occurs as laminarin (Fig. 5).

## Laminarin Contributes to Carbon Export from the Surface Ocean.

The data obtained in the Arctic and in the North Atlantic Ocean revealed laminarin in POM below the photic zone (*SI Appendix*, Fig. S15 *C* and *F*), suggesting that laminarin is part of sinking POM and thus plays an important role for carbon export to the deep ocean and the seafloor, respectively. In the Arctic, we found the highest laminarin concentrations at the Chl-a maximum between 16 and 35 m (0.46 ± 0.34 mg⋅L^−1^ [SD]) (*SI Appendix*, Fig. S15*A*), confirming that photosynthetic microalgae are the source of laminarin (34). The combined LamC:POC ratio (*SI Appendix*, Fig. S15*B*) obtained in both years was 50 ± 18% (SD), which did not significantly change with depth (*P* > 0.05, Kruskal–Wallis test) (*SI Appendix*, Fig. S15*C*), suggesting the LamC:POC ratio of the surface ocean influences deeper waters. We measured laminarin in sinking particles in the North Atlantic (Porcupine Abyssal Plain) with a marine snow catcher (35) and found it most abundant in the Chl-a maximum (∼1.0 mg⋅L^−1^ in the suspended fraction and ∼0.5 mg⋅L^−1^ in the settling particles) (*SI Appendix*, Fig. S15 *D*–*F*). The laminarin is exported in intact diatom cells sinking on their own or incorporated into marine snow particles like those shown in *SI Appendix*, Fig. S16. Its presence in sinking, diatom-containing particles (48 ± 7% [SD] LamC:POC) suggests that laminarin contributes significantly to the carbon and energy flow to the deeper ocean.

## Laminarin Is Rapidly Degraded in High Molecular Weight Dissolved Organic Matter.

The high abundance of laminarin, its rapid turnover, and ubiquitous laminarinase expression throughout the ocean indicate this molecule fuels the marine carbon cycle. Despite the high concentration of laminarin in North Sea POM, laminarin was below the detection limit in HMW-DOM that was concentrated during 8 d in 2016 from 100-L seawater on a 1-kDa ultrafiltration membrane. Although the size of laminarin allows it to be concentrated on the 1-kDa membrane, we cannot exclude the possibility that it was enzymatically degraded during the process, which would have led to laminarin loss and consequently concentrations below the detection limit. Nevertheless, this absence contrasts with axenic laboratory cultures of diatoms, in which laminarin accounts for up to 70% of the HMW-DOM being released by secretion and cell lysis (36). Absence of laminarin in marine HMW-DOM is consistent with its rapid turnover because bacterial laminarinases and laminarin uptake transporters are among the highest expressed proteins during algal blooms (5, 37). Moreover, the degradation rates of laminarin are high (1.6 to 22.0 nmol⋅L^−1^⋅h^−1^ as glucose equivalents) throughout the Atlantic (38), in particles (39), and in sediments (40) compared to other polysaccharides tested. Several studies conducted in various oceanic regions measured laminarin degradation rates of up to ∼34 nmol monomer⋅L^−1^⋅h^−1^ (*SI Appendix*, Table S2) (38–47). All these studies quantified extracellular enzymatic degradation rates of different marine polysaccharides. Moreover, among tested model polysaccharides, laminarin was the only one that was degraded everywhere (44).

Abundance of laminarin-degrading bacteria in the North Sea (34, 37), as well as the reactivity and solubility of laminarin, implies that the measured, “particulate” laminarin was protected against bacterial degradation and dissolution within the vacuoles of intact diatom cells (48). By contrast, after release of laminarin into the water, it was quickly turned over by bacteria through extracellular, enzymatic degradation, but also by direct uptake by bacterial cells without prior degradation (49). During the cruise in the Mid-Atlantic, 9% of the bacterioplankton imported laminarin directly without previous extracellular hydrolysis (45). Thus, the extracellular degradation by bacterial enzymes and the direct bacterial uptake likely results in nondetectable concentration in HMW-DOM (37). Rapid turnover of laminarin is consistent with the relatively low concentration of glucose in acid-hydrolyzed DOM obtained from different regions of the surface ocean (4).

## Discussion and Outlook

In this study, we report the substantial contribution of laminarin to the algal particulate organic carbon pool, with abundances varying with sampling time and geographic region. The diel cycle of laminarin observed in this study may affect the elemental stoichiometry of the particulate organic matter pool. The canonical mean atomic C/N Redfield ratio of marine phytoplankton is 6.6 (32), which is close to the mean of our dataset 8 ± 4, yet previous studies showed that the range of C/N values in marine microalgae can be quite large (33). Likewise, large differences in the C/N ratio were observed in different oceanic regions: In the North Pacific and Bering Sea, C/N values ranged from 3.3 to 20 (median 8.5) (50); in the North Pacific Central Gyre, from 6.9 to 48.1 (median 11.4) (51); and, in the Mediterranean Sea, from 7.8 to 40.8 (mean 14.7) (52). Our dataset ranged between 2.4 and 24 (mean 8.2 ± 4.0). North Sea samples from the Raunefjorden had the highest laminarin concentrations, with an average C/N ratio of 11.9. The synthesis of proteins, DNA, RNA, and other nitrogen-bearing molecules can be constrained by low values of dissolved inorganic N, which limits the growth of microalgae during the stationary phase of an algal bloom. Polysaccharides are produced as long as CO_2_ and light are abundant, and this continuing photosynthesis of polysaccharides may lead to elevated C/N ratios toward the end of algal blooms (33, 53–57). There may be other polysaccharides that contribute to elevated C/N values, but this hypothesis can only be accurately tested with newly developed, polysaccharide type-specific enzyme assays.

Laminarin is enzymatically readily hydrolyzed to glucose and subsequently converted into metabolic energy. The high abundance of laminarin in phytoplankton POM reported in this study agrees with acid hydrolysis experiments demonstrating that up to 90% of the monosaccharides indeed consist of glucose (58). Yet, this high amount of glucose is not detected in acid-hydrolyzed DOM extracted from seawater (59), despite active secretion, lysis, and grazing of phytoplankton cells. Taken together, these results support the extensive degradation of laminarin by secreted laminarinases and through direct bacterial uptake. Given the high proportion of laminarin in diatoms and the role that they play in carbon export, laminarin flux may be of global relevance. The amount of DOM available to heterotrophic bacteria from algal particulate organic carbon is estimated to be up to 50% (60). Considering a marine carbon fixation rate of up to 3 mg C⋅m^−3^⋅d^−1^ (or ∼10 nmol⋅L^−1^⋅h^−1^) (61) and assuming that 37% of this is laminarin with an average DP of 22, the molecule’s formation rate in the dissolved organic carbon pool would be up to ∼4.4 nmol⋅L^−1^⋅h^−1^. It is produced by algae during the day and devoured by selfish, sharing, or scavenging bacteria when algal cells lyse. The measured hydrolysis rates of 0.1 to 34 nmol⋅L^−1^⋅h^−1^ (*SI Appendix*, Table S2) indicate a fast enzymatic degradation of laminarin and underscore the relevance of this molecule in the marine carbon cycle, especially in productive regions rich in diatoms and other laminarin-containing algae, such as *Phaeocystis* spp. Our observations also suggest that laminarin is temporarily preserved from degradation in POM, providing a pathway for laminarin export in sinking diatom cells that are part of marine snow. How the temporally variable LamC:POC ratio in phytoplankton POM relates to carbon export and regulates marine ecosystem properties, such as growth, behavior, and composition of higher trophic species, are important questions, which can now be addressed with recently introduced enzymatic assays that enable identification and quantification of complex glycans.

## Methods

### Sites and Sample Collection.

The entire dataset comprises samples from six oceanic regions (Fig. 1). They were taken during eight separate cruises or campaigns (*SI Appendix*, Table S1). The filtered volume was logged for every sample. Prior to sampling, the glass fiber filters were combusted for 4 h at 450 °C to remove carbon contamination. After sampling, the filters were wrapped in precombusted aluminum foil and kept frozen at −20 °C until further processing. Blank samples were taken by filtering 10 L of MilliQ-H_2_O. The North Sea was sampled during two campaigns in spring 2016 (from March to June) and 2017 (from January to May) at the long-term ecological research site (LTER) “Helgoland Roads” at the island of Helgoland in the German Bight (Fig. 1). The sea surface samples were collected in 50-L bottles (Nalgene), and the water was filtered sequentially through 10-µm, 3-µm polycarbonate (PC) and 0.2-µm polyethersulfone filters (Whatman). An extra 142-mm glass fiber filter, grade F (GF/F) sample (pore size of ∼0.7 µm; Whatman) was collected during the 2017 campaign. Additionally, 100 L of high molecular weight dissolved organic matter (HMW-DOM) were concentrated using a 1-kDa membrane and tangential ultrafiltration (Sartorius). In order to investigate different size fractions, we took additional samples on GF/F (pore size of ∼0.7 µm; Whatman) and glass fiber filters, grade D (GF/D, pore size of ∼3 µm; Whatman) during another campaign on Helgoland in spring 2018. The Arctic was sampled during the two cruises PS99.2 in 2016 (from June to July) and PS107 in 2017 (from July to August) at eight different stations of the LTER “HAUSGARTEN” (Fig. 1) by using GF/D filters at a pore size of ∼3 µm (Whatman). Different water depths were sampled in situ by deploying several large volume water transfer systems (WTS-LVs) (McLane) at the same time. The pumps were attached to the cable of the rosette water sampler and filtered water over the duration of 60 min. The Mid-Atlantic was sampled during the Atlantic Meridional Transect 22 (AMT) cruise in 2012 (from October to November) at 23 stations. Sea surface water was collected using Niskin bottles in a rosette water sampler. This water was sequentially filtered through 10- and 3-µm polycarbonate filters (Whatman). The Canary upwelling system was sampled during the POS508 cruise in 2017 (from January to February) at five stations. Two sea surface samples were taken by the rosette water sampler, and three were taken by using the ship’s onboard flowing seawater system; all were filtered through GF/D filters (Whatman). The Peru upwelling system was sampled during the M138 cruise in 2017 (from June to July) at nine stations. The rosette water sampler was used to collect sea surface water, which was filtered through GF/D filters (Whatman). The North Atlantic was additionally sampled during the DY077 cruise in April 2017 at three stations in the area of the Porcupine Abyssal Plains. Sea surface water was sampled by using the ship onboard flowing seawater system. Additionally, a large volume water sampler (OSIL) was used at different depths to fractionate suspended and settling particle samples (marine snow). Settling particles were sedimented by letting the snow catcher stand upright for 9 to 31 h on deck after it had been deployed and returned to the surface. Later, the suspended particles in the top section were slowly drained, and the remaining volume of the bottom section, including the settling particles, was subsequently recovered. Both fractions were filtered through GF/D filters (Whatman).

### HMW-DOM Sample Processing.

The ∼45-h concentration of the 100-L sample via tangential ultrafiltration (Sartorius) on a 1-kDa membrane resulted in a 0.5-L volume, which was frozen at −20 °C for later processing. The sample was then thawed and further concentrated to a volume of ∼25 mL using an Amicon ultrafiltration stirred cell (Merck-Millipore) with a 1-kDa membrane. Afterward, it was desalted by dialysis using a 1-kDa membrane (Spectra/Por). The retentate material was then frozen and freeze-dried (Labogene). This procedure yielded several milligrams of lyophilized material, which could be again dissolved for further analyses.

### Polysaccharide Quantification.

The sample preparation steps, including extraction, enzymatic hydrolysis, and the final measurement of laminarin hydrolysis products via an adapted protocol of the *p*‐hydroxybenzoic acid hydrazide (PAHBAH) reducing sugar assay, were performed according to Becker et al. (17) and Becker and Hehemann (18). Amylose was quantified in the exact same way using a commercial amylase (Sigma). Total carbohydrates were determined via mild acid hydrolysis using 1 M HCl in a sealed glass ampoule for 24 h at 100 °C, followed by neutralization by speed-vac, resuspension in Milli-Q water, and quantification via PAHBAH, phenol sulfuric assay (PSA), or high performance anion exchange chromatography (HPAEC). Strong acid hydrolysis was carried out for 2 h at 25 °C using 12 M H_2_SO_4_, followed by 3 h at 100 °C and 1.2 M H_2_SO_4_. PSA was performed according to an adapted protocol of Dubois et al. (62).

### Effect of Laminarin Molecular Weight and Structure on the Quantification with the Biocatalytic Assay.

The laminarin carbon content was calculated based on the assumption of an average laminarin molecule with DP 22 and DB 10, which consists solely of glucose monomers but takes the decreased number of hydrogen and oxygen atoms into account that the glycosidic linkages account for, resulting in a calculation factor of 0.44. In comparison, a doubled DP 44 does not change the calculation factor significantly. We previously tested whether the molecular weight of laminarin makes a difference by using laminarins with different molecular weight and degree of branching from *Eisenia bicyclis* and *Laminaria digitata* for calibration and found almost identical calibration curves with the biocatalytic assay (17, 18).

### Product Analysis Using High Performance Anion Exchange Chromatography with Pulsed Amperometric Detection.

To exemplify the function of our enzymatic assay on an environmental sample, we hydrolyzed a sample stepwise and detected the resulting products via HPAEC with pulsed amperometric detection (PAD). The sample extract from Helgoland at April 27, 2017 was first hydrolyzed with 100 nM FbGH30 for 25 min at 37 °C, and then FaGH17A was applied in the same manner. Between each digestion, the reaction was stopped by boiling the sample for 5 min at 100 °C; precipitated protein was removed by filtration through 0.2-µm centrifuge filters (Costar Spin-X; Corning), and an aliquot was taken.

Samples were applied on an ICS-5000+ (Dionex) with electrochemical detection on a gold working electrode and a pH reference electrode (Ag/AgCl) according to Unfried et al. (37). Separation was attained by using a Dionex CarboPac PA100 analytical column at 35 °C. Glucose (Sigma), laminaribiose, laminaritriose, laminaritetraose, and laminaripentaose (all from Megazyme) were used as reference.

### Particulate Organic Carbon and Nitrogen Measurement.

For all but the Mid-Atlantic samples, glass fiber filters were used for sampling. The same glass fiber filter (GF/D or GF/F) was used for both the laminarin and particulate organic carbon and nitrogen measurements. After punching out defined pieces of the filter in triplicate, the pieces were subjected to an acidic atmosphere with concentrated HCl for 24 h in a desiccator to remove inorganic carbon. They were then dried for 24 h at 60 °C and afterward packed in combusted tin foil. The carbon and nitrogen quantification was performed by an elemental analyzer (vario MICRO cube; Elementar Analysensysteme) using sulfanilamide for calibration. For the Mid-Atlantic samples, which were filtered on polycarbonate filters, this procedure could not be applied.

### NMR Spectroscopy.

Freeze-dried POC filter residues (5 to 250 mg) were dissolved in 500 µL of deuterated water (D_2_O) or deuterated dimethyl sulfoxide (DMSO). NMR spectra were obtained on a Bruker AVANCE 600 spectrometer (Bruker Biospin, Rheinstetten, Germany) and recorded at 600.2 MHz for ^1^H and 150.9 for ^13^C nuclei, using a 5-mm PABBI or CPTCI probe head and standard Bruker pulse programs. Trimethylsilylpropanoic acid was used as internal standard for quantification. Signals were assigned as described by Kim et al. (30) and Størseth et al. (31). Only D_2_O could completely dissolve the sample; therefore, samples (5 mg⋅mL^−1^) were dissolved in this solvent for laminarin quantification. Quantification was achieved via integration of the well-resolved H-6 proton. Structural characterization and shift comparison with literature values was done of the sample in deuterated DMSO. ^13^C chemical shifts were calibrated using the methyl resonances of DMSO at 39.5 ppm.

### Protein Carbon and Lipid Carbon Quantification.

Total protein and total lipid extractions were conducted on pieces from the same glass fiber filters that were used for laminarin and POC measurements. After punching out defined pieces of the filter in triplicate, the pieces were extracted according to an adapted protocol of Slocombe et al. (63). After adding 6% trichloroacetic acid to the filters and vigorous vortexing, the samples were incubated at 95 °C for 15 min. Precipitated protein and the filter material were centrifuged at 15,000 × *g* for 20 min at 4 °C, and the supernatant was discarded. Then, 1 mL of Lowry reagent was added to the pellet and the filter, followed by vigorous mixing. The rest of the protocol was performed according to the manufacturer’s manual of the Total Protein Kit (Sigma-Aldrich), which is based on Peterson’s modification of the original Lowry assay (64, 65).

Total lipids were extracted by acid hydrolysis with 1 M HCl in methanol, as described by Becker et al. (66). Filters were placed in a Teflon extraction vial containing the acid mix and combusted sand. The mixture was sonicated two times for 20 min, and hydrolysis was performed overnight (approx. 16 h) at 70 °C. Afterward, the extraction vial was sonicated two more times for 20 min and centrifuged for 10 min at 400 rpm, and the supernatant was poured in a separatory funnel. The filters were then extracted three more times by sonication for 20 min with 5:1 (v:v) dichloromethane:methanol and centrifuged, and supernatants were combined in the separation funnel. Deionized water was added, and the organic phase was collected after phase separation. The aqueous phase was then washed two times with dichloromethane and the combined organic phase three times with deionized water. The organic phase was dried under a stream of N_2_ and transferred to a preweighed 2-mL vial. The total lipid weight was determined by five successive measurements on a microscale balance (Mettler-Toledo). The mean relative error was determined to be 3.8%. A blank filter was extracted following the same procedure to check for potential nonlipid contamination. Total protein and lipid concentrations were converted to carbon equivalents, assuming conversion factors of 0.50 and 0.75, respectively (67–71).

### Complementary Data.

The Helgoland Roads LTER time series provides Chl-a and temperature data on a weekday basis (72). Multiwavelength spectrofluorometric analysis (bbe Moldaenke) provided an estimate of the relative pigment contributions of diatoms, chlorophytes, cryptophytes, and cyanobacteria (20) during this campaign. Throughout both Arctic cruises, fluorescence measurements were conducted on the rosette water sampler that was deployed together with the in situ pumps (73, 74). The measurements were calibrated with HPLC-based chlorophyll data (75). These data are partly already accessible via the public database Pangaea (https://www.pangaea.de) (76). The study uses also Chl-a and temperature data from Gavin Tilstone/Plymouth Marine Laboratory/Oceans 2025 project S01 Atlantic Meridional Transect, provided by the British Oceanographic Data Centre and funded by the Natural Environment Research Council (https://www.bodc.ac.uk/).

Extracellular enzymatic activities (EEAs) of polymeric substrates, especially via endo-active enzymes, yield an estimate on the potential hydrolysis rates of laminarin and other polysaccharides by a bacterial community. This technique was developed by Arnosti and colleagues and makes use of fluorescently labeled polysaccharides (77, 78). During hydrolysis, the fluorescent tags stay attached to the substrates, and the respective hydrolysis rates can be determined by the measurement of a decrease in substrate size during incubation via size exclusion chromatography.

The maximum theoretical laminarin formation rate in the dissolved organic carbon pool was calculated by translating the maximum carbon fixation rate of ∼3 mg C⋅m^−3^⋅d^−1^, that was found by Behrenfeld and Falkowski (61), to 10.4 nmol C⋅L^−1^⋅h^−1^.This value was multiplied by 0.5, which refers to up to 50% of the carbon that is being made available to heterotrophic digestion from algal particulate organic carbon, that was estimated by Azam et al. (60). This value was again multiplied by 0.37, the 37% fraction of this carbon pool constituted by laminarin. Based on that and by assuming a carbon mass contribution to an average laminarin molecule of ∼44% (DP 22/DB 10), one can calculate a formation rate of up to ∼4.4 nmol⋅L^−1^⋅h^−1^.

### Statistical Analysis.

Statistical analysis was carried out by using the R environment v.3.4.3 (R Core Team 2017, https://www.r-project.org). Linear models were fitted to the investigated variables. Significant differences in the nonnormal distributions of values among groups were tested by the Kruskal–Wallis test. *P* values below 0.05 were considered statistically significant. The confidence interval for the median of nonnormal data was calculated via bootstrapping with 1,000 replications (79).

### Data Availability.

Environmental data used in this manuscript will be made accessible via the public database Pangaea (https://www.pangaea.de).

## Supplementary Material

Supplementary File

Supplementary File

Supplementary File
