# Supplementary material for: Laminarin is a major molecule in the marine carbon cycle
Source: Proc Natl Acad Sci U S A. 2020 Mar 13;117(12):6599–607. doi: 10.1073/pnas.1917001117 (PMC7104365; doi:10.1073/pnas.1917001117)
Supplement: Supplementary File [file pnas.1917001117.sapp.pdf]

SI

Comparison of new enzyme with classic sugar quantification method

In order to put our results based on the enzymatic glycan assay into perspective with literature data, we first validated established techniques for the quantification of monomeric hydrolysates using pure laminarin and environmental samples as reference materials. Three standard techniques for quantifying monomers derived from either enzymatic or acidic hydrolysis, p-hydroxybenzoic acid hydrazide (PAHBAH) assay, high performance anion exchange chromatography analysis (HPAEC) and the phenol sulfuric acid assay gave similar values (See SI Appendix, Fig. S3). By contrast, the acid hydrolysis method is non-selective and thus not suitable for quantifying monosaccharides derived from specific glycan structures as it hydrolyzes also other polysaccharides than laminarin (See SI Appendix, Fig. S4).

Although the acid method gave higher values than laminarinases, it may still underestimate the total carbohydrate content. To verify these findings and to test to what extend enzymes may underestimate or overestimate carbohydrate content we amended the laminarin containing environmental samples with additional, known concentration of glucose or laminarin and quantified the glucose signal again with all three methods of detection versus acid or enzymes for all amendments. In reactions with enzymes, we found that the median glucose and laminarin concentrations decreased to 99% and 87% compared to what was previously added to the sample from the Canary upwelling and the North Sea, respectively (See SI Appendix, Fig. S5). This shows that, although enzymes may underestimate the laminarin concentration, we found no evidence for overestimation. The reduction in spiked signal was more sample/region dependent in the acid treated samples. The acid method produced values that were lowered to 93%, 82% and 68% in the spiked samples from the North Atlantic, the Canary upwelling and the North Sea, respectively (See SI Appendix, Fig. S5).

Acid catalyzed side reactions between aldehydes and amines may decrease measured carbohydrate concentrations in the ocean. Multiple mechanisms lead to sugar losses: sample

preparation (e.g. during lyophyllization), incomplete hydrolysis due to stability of the glycosidic bond, monomer destruction by acid, side reactions such as Amadori rearrangement and the Maillard reaction between amines from proteins and aldehyde groups of sugars. To test if the sugar losses occurred by sample preparation we prepared pure glucose in seawater as test sample. We could recover about 100% of the glucose with enzyme and acid hydrolysis suggesting that our analysis pipeline of hydrolysis, lyophillization followed by three different detection methods did not cause the losses (See SI Appendix, Fig. S6). Likewise acid induced monomer destruction did likely not account for the losses. The same experiment with laminarin as test substance gave again 100% of the signal, hence incomplete hydrolysis of laminarin could be ruled out (See SI Appendix, Fig. S6). To test if acid catalyzed reactions caused the losses we added increasing concentrations of bovine serum albumin (BSA) to glucose and found similar losses with acid as with environmental samples (See SI Appendix, Fig. S6). However, especially combined with reducing sugar assays the acid hydrolysis method may also sometimes lead to overestimated values of carbohydrates as all reducing compounds add to the signal. High concentrations of BSA significantly decreased the glucose recovery with the mild acid hydrolysis protocol (24 h at 100°C using 1 M HCl). The laminarin recovery decreased significantly with BSA with the commonly used strong acid hydrolysis protocol (2 h at 25°C using 12 M H_2SO_4 followed by 3 h at 100°C and 1.2 M H_2SO_4). These results suggest that acid catalyzed Maillard reactions between sugar and proteins decrease carbohydrate recovery. This effect is likely dependent on the type of poly- and monosaccharide analyzed and dependent on the amount of reactive amines in the sample.

Supplementary discussion

Different filters were used in the eight datasets. Arctic datasets, the North Atlantic, Canary upwelling and Peru upwelling dataset made use of glass fiber filters with an approximate pore size of 3 µm. In contrast, the POC measurements of the Helgoland 2017 dataset were

based on glass fiber filters with an approximate pore size of 0.7 µm. The Mid-Atlantic and Helgoland 2016 dataset relied on polycarbonate filters, where POC determination was not applicable. POC is usually sampled and measured on 0.7 µm glass fiber filters. In order to investigate the possible variations in the different size fractions, we took additional samples during another campaign on Helgoland in May 2018. There, we took two times six samples on both types of glass fiber filters. On average, the POC content of the >3 µm fraction was 16±5% (SD) lower compared to the >0.7 µm fraction. This means that the average value of 37±17% laminarin per POC, which is primarily based on measurement of ~3 µm micrometer filters, can change up to 5%, depending on the pore sizes of these two filter types.

We described all of our samples as POC samples irrespective of the two different pore sizes. In general, the term particulate organic matter is poorly defined and investigations on the POC yield in different size fractions are scarce. Comparisons were made on mineral material but not in natural seawater. Gardner et al. elaborated on this topic and concluded that more experiments have to be made and that the current boundaries between particulate and dissolved organic matter are arbitrary (1, 2). Koike et al. (3) found that >95% of the POC up to a size of 1 µm is non-living organic matter, in which we would not expect to detect laminarin.

For the North Sea 2017 dataset, we tested 0.7 µm glass fiber filters using a single filtration step, which reproduced the combined laminarin signal of the 10 and 3 µm polycarbonate filters (See SI Appendix, Fig. S7A). This result was also illustrated by the slopes of the linear correlations (See SI Appendix, Fig. S7A). The laminarin content on the glass fiber filter (GF/F), the 10 µm, and the combined 10 and 3 µm fraction on polycarbonate filters (PC) increases with similar slope. In contrast, the 3 µm fraction did not increase and barely correlates with chlorophyll a. This result indicates not only that the laminarin increase during the bloom was driven by larger microalgae, but also that different filters can be used for quantitative laminarin analysis. No laminarin was detected in the 0.2 µm fraction, which is expected to contain mostly bacteria, suggesting laminarin is produced by larger microalgae.

The sequentially filtrated 100 L were further concentrated using a 1 kDa membrane. In the resulting HMW-DOM of the eight samples that were chosen from the entire period of the sampling campaign, we were not able to detect laminarin, meaning the hydrolyzed laminarin samples yielded the same low signal as the non-hydrolyzed blanks. Adding known amounts of laminarin to these samples was used to confirm the validity of the assay. We could detect the added laminarin molecules confirming the absence of potential enzyme inhibition.

Both Arctic datasets exhibit a similar pattern of laminarin and POC distribution, with higher values at the depth of the chlorophyll a maximum (between 16 and 35 m) and lower values at the surface and the deeper water layer around 300 m (See SI Appendix, Fig. S15A,B). The cruise in 2016 took place earlier in the year (from June to July) and yielded on average higher values at the chlorophyll a maximum and lower values in the deep. Compared to this result, the data from July to August 2017 shows a more even distribution over depth, with lower values in the chlorophyll a maximum, but higher values at the surface and in the deep. This result might indicate that at this later time in the year, the bloom was already in a more progressed stadium compared to 2016.

References

1. J. H. Sharp, Improved analysis for “particulate” organic carbon and nitrogen from seawater. *Limnol. Oceanogr.* **19**, 984–989 (1974).
2. W. D. Gardner, M. J. Richardson, C. A. Carlson, D. Hansell, A. V Mishonov, Determining true particulate organic carbon: bottles, pumps and methodologies. *Deep Sea Res. Part II Top. Stud. Oceanogr.* **50**, 655–674 (2003).
3. I. Koike, H. Shigemitsu, T. Kazuki, K. Kazuhiro, Role of sub-micrometre particles in the ocean. *Nature* **345**, 242–244 (1990).
4. K. L. Terry, J. Hirata, E. A. Laws, Light-limited growth of two strains of the marine diatom Phaeodactylum tricornutum Bohlin: Chemical composition, carbon partitioning and the diel periodicity of physiological processes. *J. Exp. Mar. Bio. Ecol.* **68**, 209–227 (1983).
5. A. Alderkamp, *et al.*, Dynamics in carbohydrate composition of Phaeocystis pouchetii colonies during spring blooms in mesocosms. *J. Sea Res.* **55**, 169–181 (2006).
6. J. A. Furgal, W. D. Taylor, R. Smith, Environmental control of photosynthate allocation in the phytoplankton of Georgian Bay (Lake Huron). *Can. J. Fish. Aquat. Sci.* **55**, 726–736 (1998).

7. T. R. Parsons, K. Stephens, J. D. H. Strickland, On the Chemical Composition of Eleven Species of Marine Phytoplankters. *J. Fish. Res. Board Canada* **18**, 1001–1016 (1961).

8. J. I. Hedges, *et al.*, The biochemical and elemental compositions of marine plankton: A NMR perspective. *Mar. Chem.* **78**, 47–63 (2002).

9. C. Nelson, *et al.*, Comprehensive functional characterization of the glycoside hydrolase family 3 enzymes from Cellvibrio japonicus reveals unique metabolic roles in biomass saccharification. *Environ. Microbiol.* **19**, 5025–5039 (2017).

Supplementary Figures

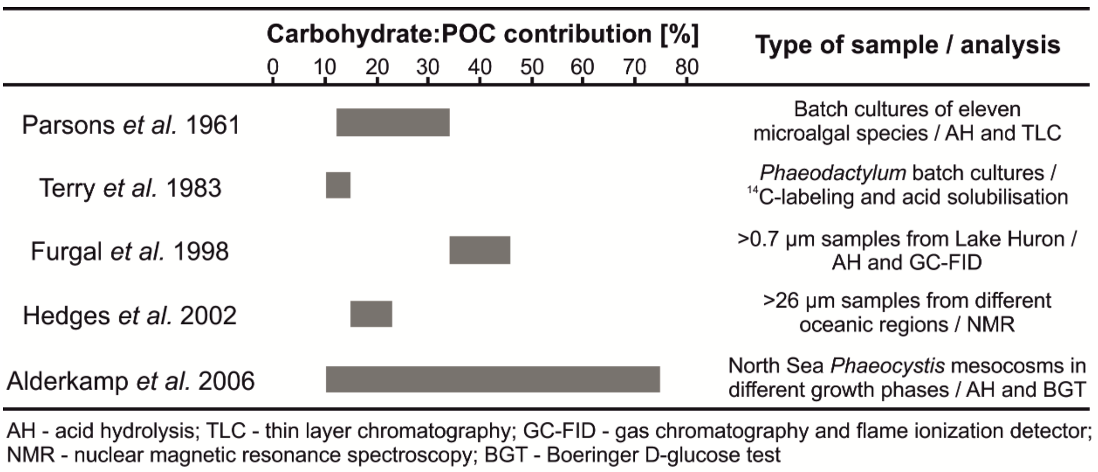

Fig. S1: Representative comparison of carbohydrate contribution to particulate organic carbon (POC) in microalgal batch cultures, mesocosm experiments and environmental phytoplankton samples (4–8)

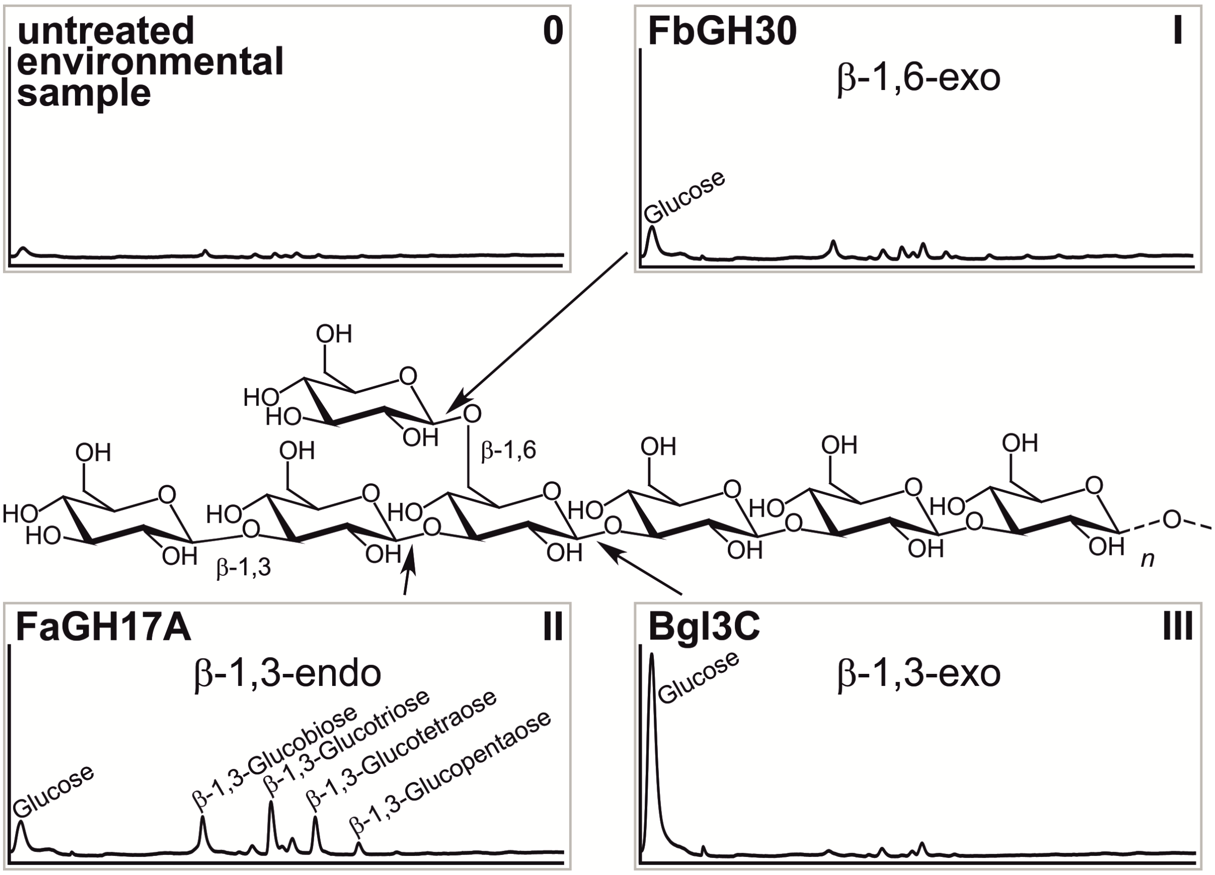

Fig. S2: Sequence of enzymatic dissection of organic matter enables quantification of laminarin. Laminarin is a β-glucan composed of a β-1,3-glucose backbone with β-1,6 linked glucose side chains. Step 0: Liquid chromatogram of an untreated sample taken on April 27, 2017, near the island of Helgoland. The three enzymes applied in steps I to III belong to the glycoside hydrolase families GH30, GH17 and GH3 respectively; enzymes GH30 and GH17 were used in the analytic assay applied to marine samples. Step I: The GH30 removes the β-1,6-linked glucose side chains. Step II: The GH17 cleaves the β-1,3-linked backbone into oligosaccharides with a degree of polymerization (DP) ≥ 2. Optional step III: The reaction can be completed by conversion of the resulting oligosaccharides into glucose (DP 1) by adding a GH3 enzyme (9).

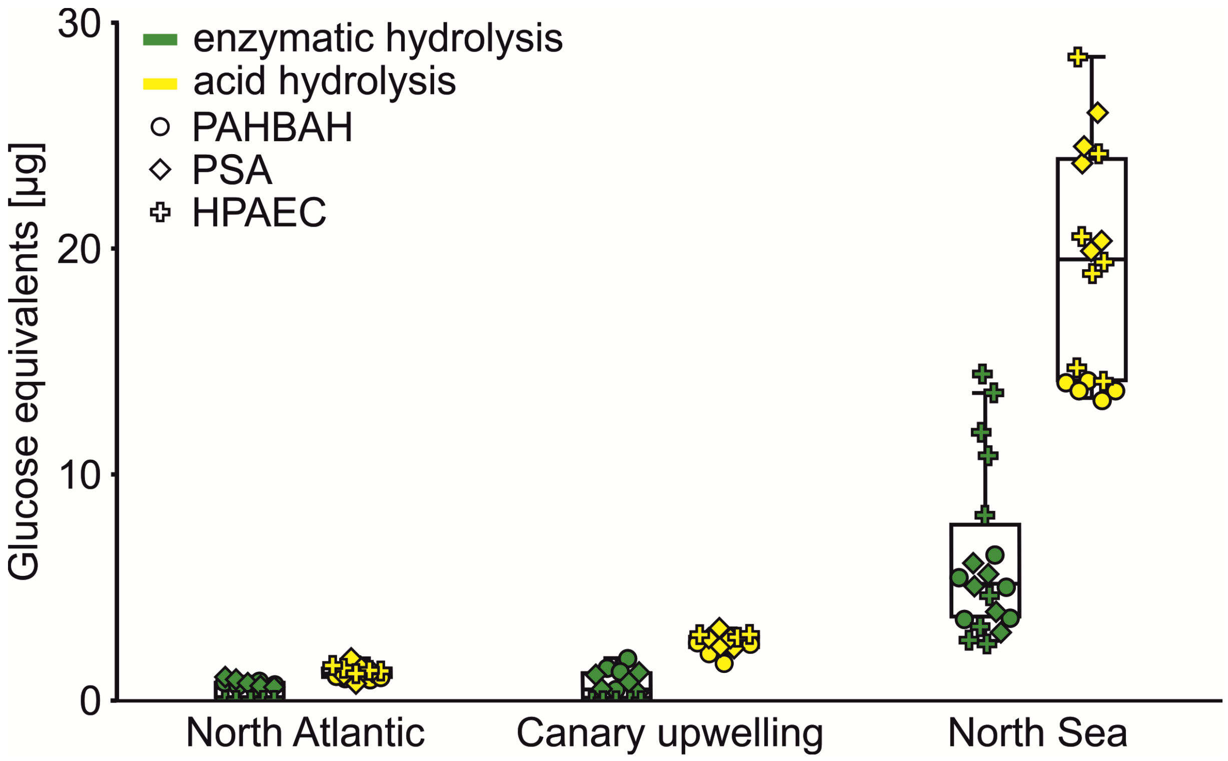

Fig. S3: Comparison of of three sugar detection methods. Environmental samples originated from the North Sea (Sample ID: H280317), the North Atlantic (Sample ID: 1_Surf) and the Canary upwelling (Sample ID: POS508_1). The PAHBAH reducing sugar assay, the phenol sulfuric assay (PSA) and high performance anion exchange chromatography (HPAEC) was used for quantification.

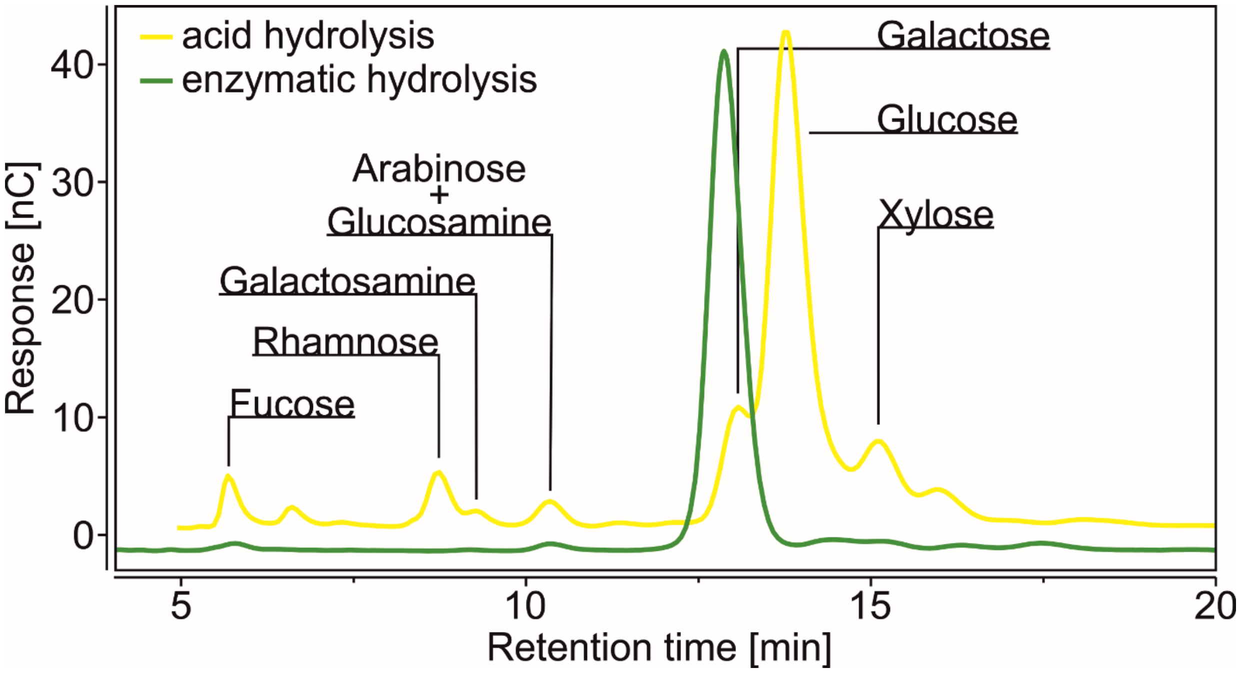

Fig. S4: Comparison of enzyme and acid hydrolysis derived monosaccharides in a native North Sea sample. The North Sea sample (Sample ID: H280317) was analyzed by high performance anion exchange chromatography with pulsed amperometric detection (HPAEC-PAD) according to Engel and Händel 2011, and the respective monosaccharides as reference standards. The offset of the second chromatogram is 1 min on the x-axis and 2 nC on the y-axis.

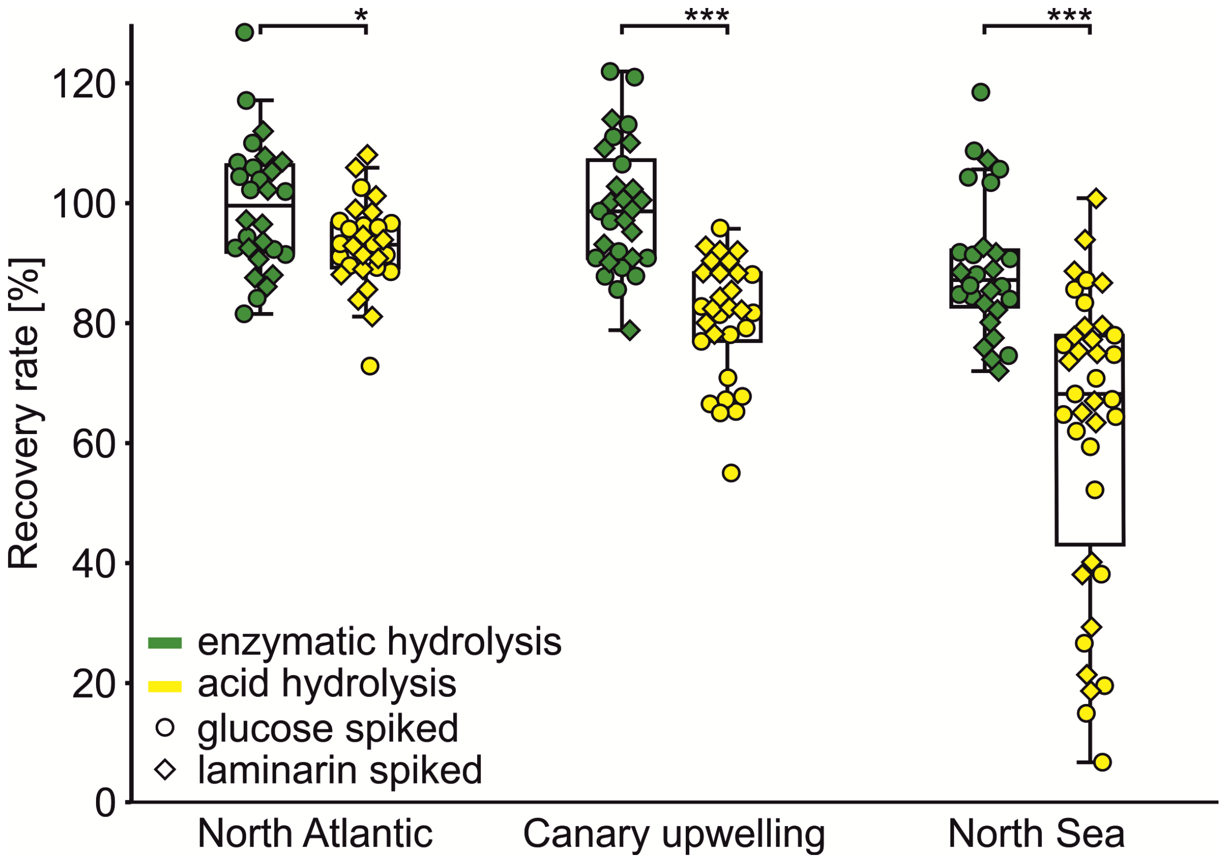

Fig. S5: Comparison of the recovery rates of enzyme and acid hydrolyzed samples. Environmental samples from the North Sea (Sample ID: H280317), the North Atlantic (Sample ID: 1_Surf) and the Canary upwelling (Sample ID: POS508_1) were spiked with different amounts of glucose and laminarin. The PAHBAH reducing sugar assay, the phenol sulfuric acid assay (PSA) and high performance anion exchange chromatography (HPAEC) was used for quantification. Mild acid hydrolysis was performed for 24 hours at 100°C using 1 M HCl. Laminarin water extraction and enzymatic hydrolysis were done for 1 h at 60°C and for 30 min at 37°C, respectively. Significant deviations from the comparison group were tested using the Kruskal-Wallis test (*, $P<0.05$; ***, $P<0.001$).

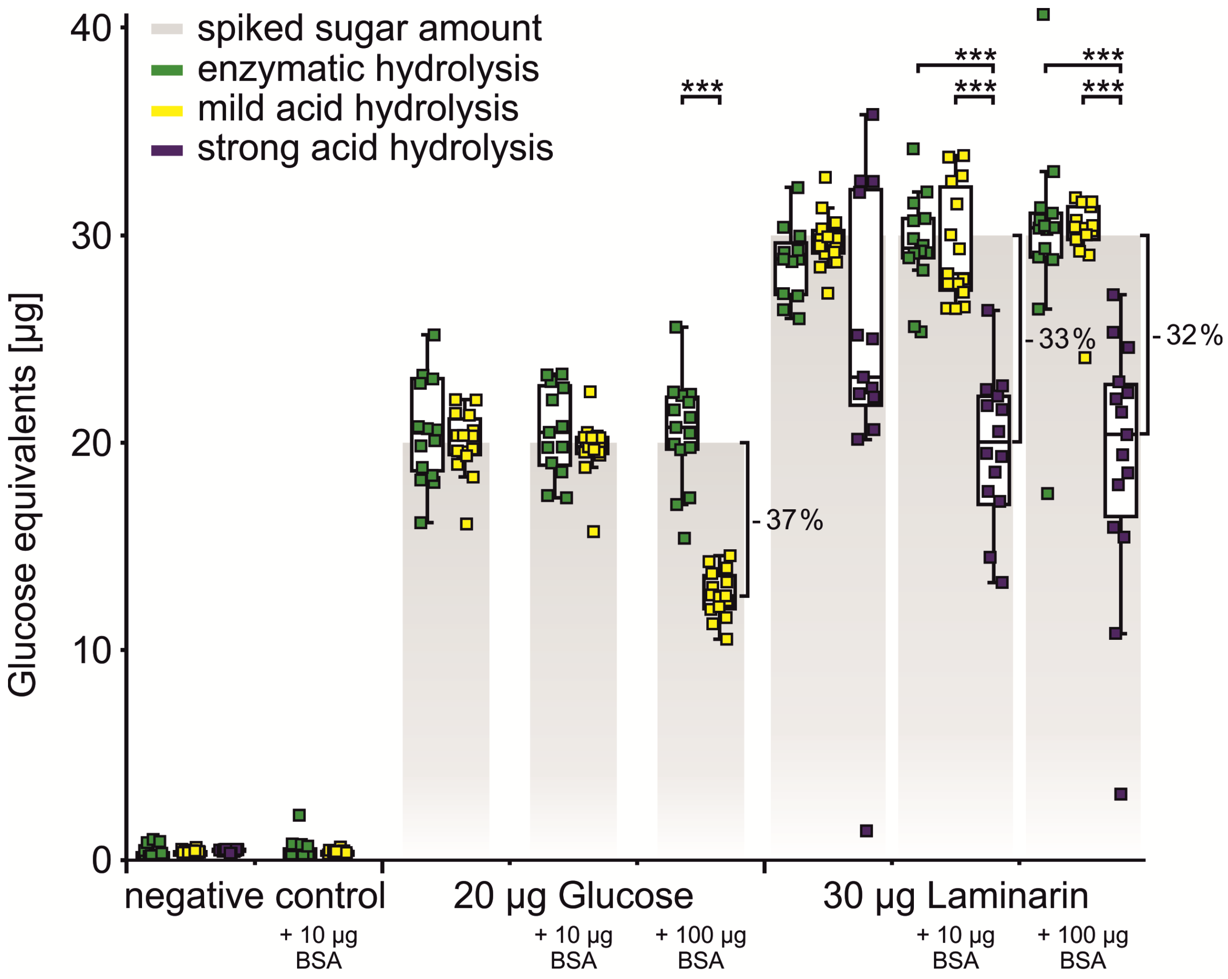

Fig. S6: Comparison of the recovery rates of enzyme and acid hydrolyzed samples based on mock samples. Samples were spiked with different amounts of glucose, laminarin and bovine serum albumin (BSA). Mild acid hydrolysis was performed for 24 hours at 100°C using 1 M HCl. Strong acid hydrolysis was carried out for 2 h at 25°C using 12 M H_2SO_4 followed by 3 h at 100°C and 1.2 M H_2SO_4. Laminarin water extraction and enzymatic hydrolysis were done for 1 h at 60°C and for 30 min at 37°C, respectively. Significant deviations from the comparison group were tested using the Kruskal-Wallis test (***, P<0.001).

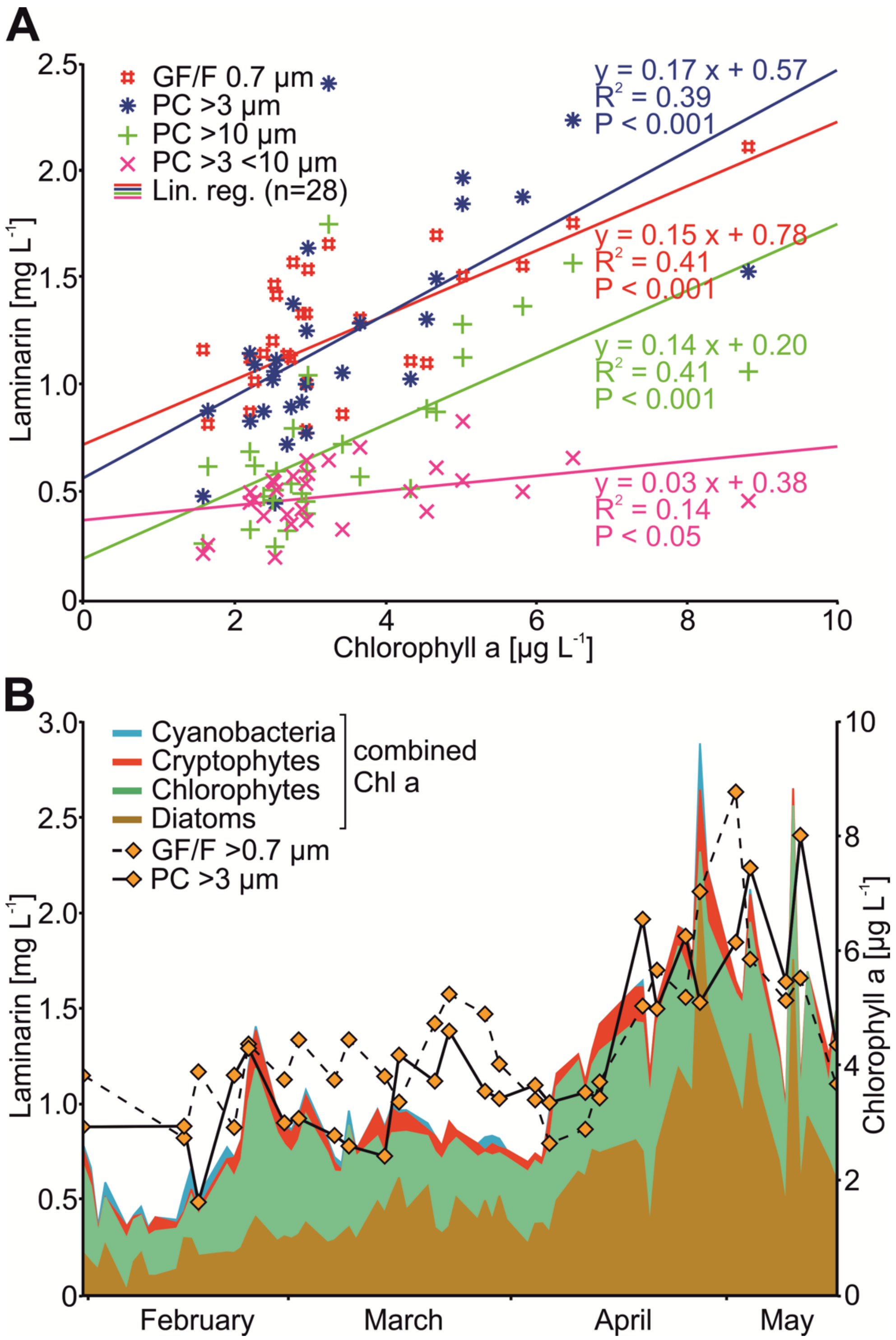

Fig. S7: Laminarin and chlorophyll a comparison on different filter fractions during a phytoplankton spring bloom in the North Sea 2017. (A) Linear regression was applied to the laminarin and chlorophyll a signal relationship of all filter fractions. **(B)** The combined signal from sequentially applied polycarbonate filters (PC) with pore sizes of 10 and 3 µm was compared to the signal of the glass fiber filters (GF/F) with a pore size of 0.7 µm, which was used without prefiltration.

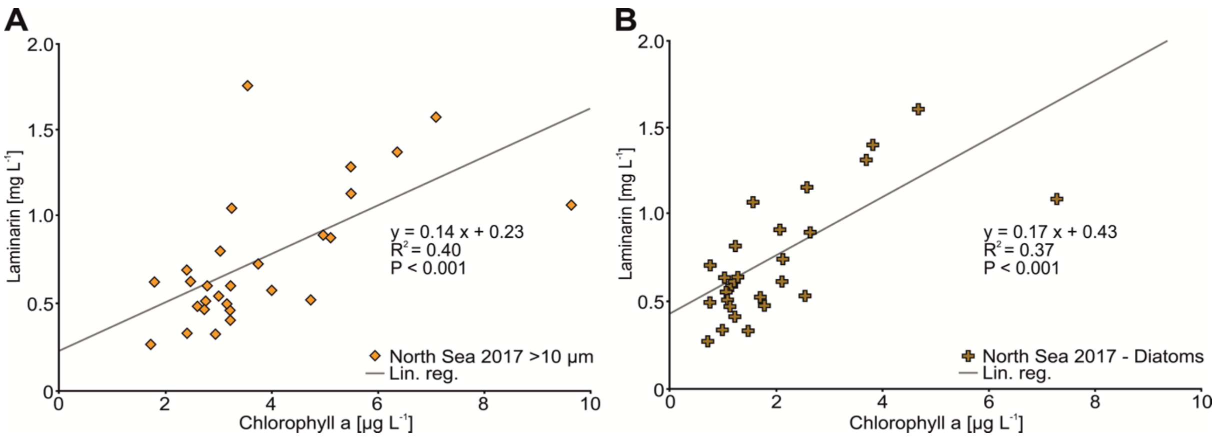

Fig. S8: Correlations between different laminarin and chlorophyll a signals. (A) Linear regression was applied to the chlorophyll a vs. laminarin (>10 µm fraction) signal relationship during the North Sea 2017 phytoplankton bloom. **(B)** Linear regression was applied to the relationship between the laminarin (>10 µm fraction) and chlorophyll a signal from diatoms during the North Sea 2017 phytoplankton bloom.

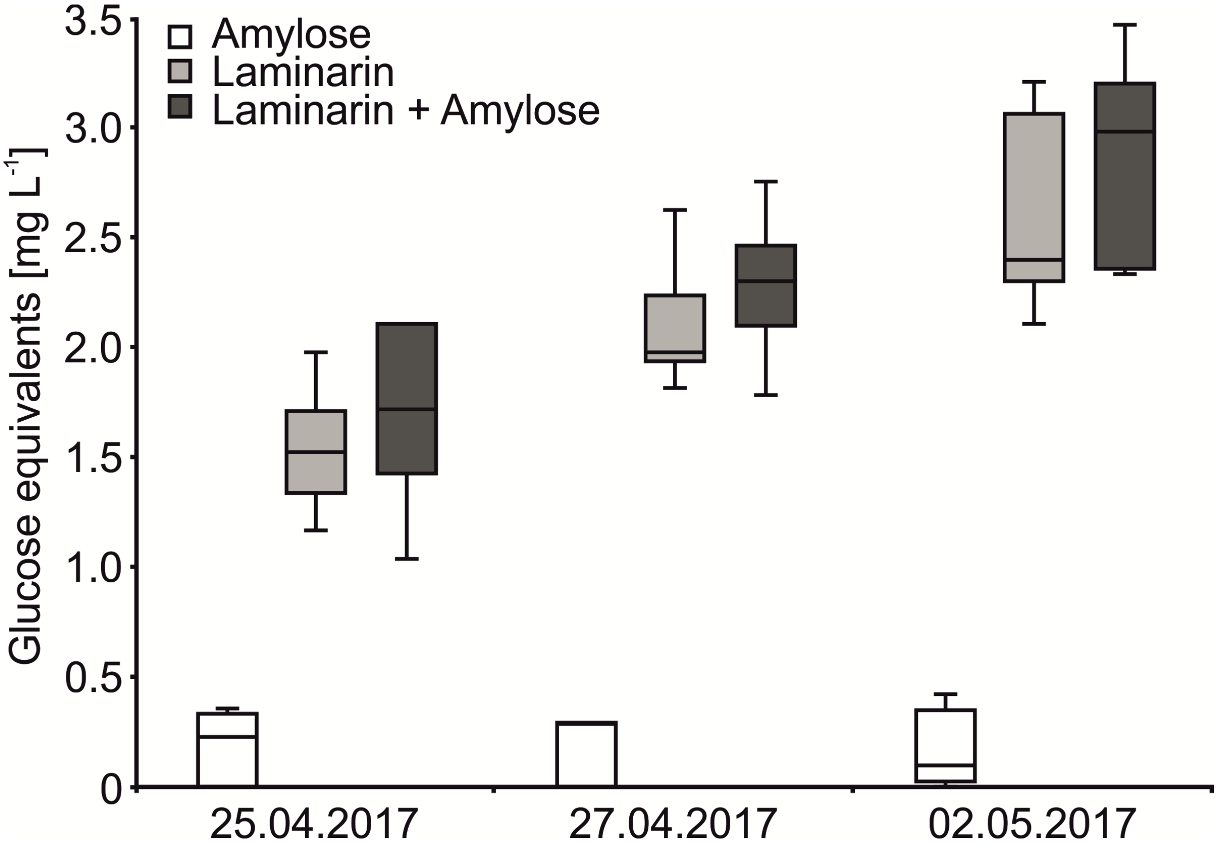

Fig. S9: Comparison of laminarin and amylose content of a selected subsample from three days during the Helgoland 2017 campaign.

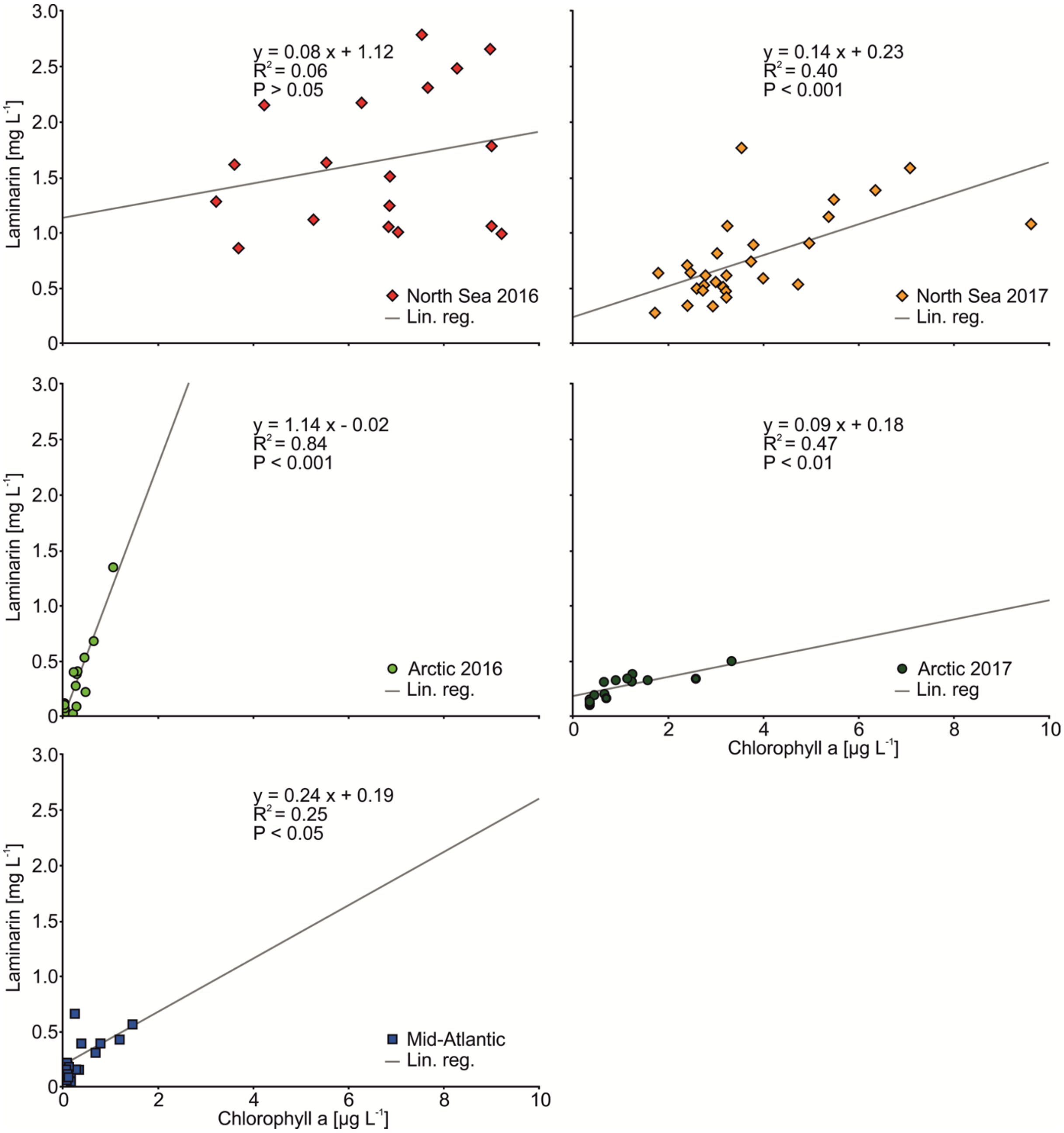

Fig. S10: Comparison between laminarin and chlorophyll a concentrations in separate oceanic regions. Linear regression was applied to the laminarin to chlorophyll a relationship.

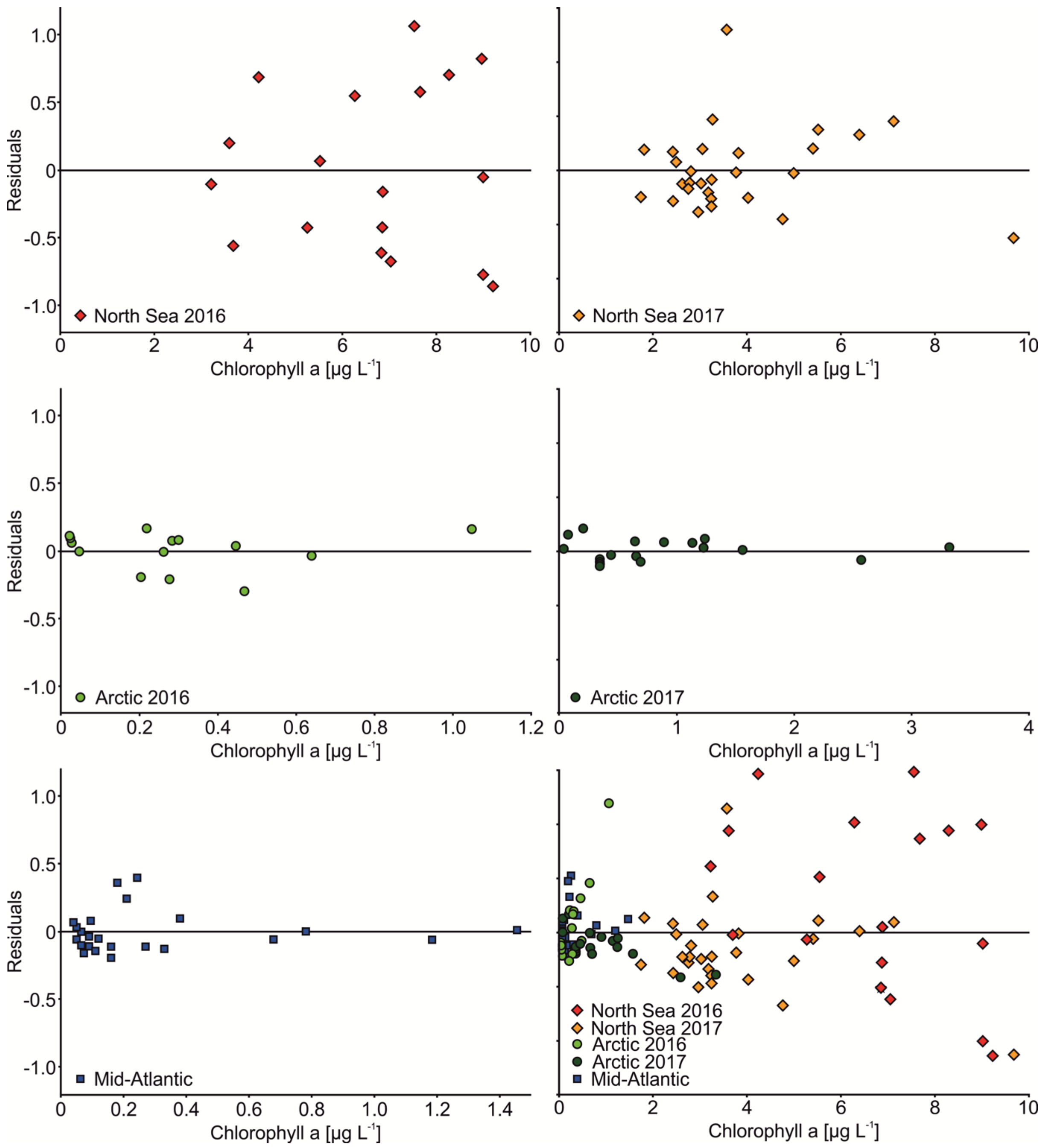

Fig. S11: Residual contributions of the linear models of laminarin to chlorophyll a content. Linear regression was applied to the laminarin to chlorophyll a relationship. The scatter plots depict the distributions from each linear regression model applied to those regions where chlorophyll a was measured individually. The integrated scatter plot (bottom right) comprises the residuals from the overall linear model.

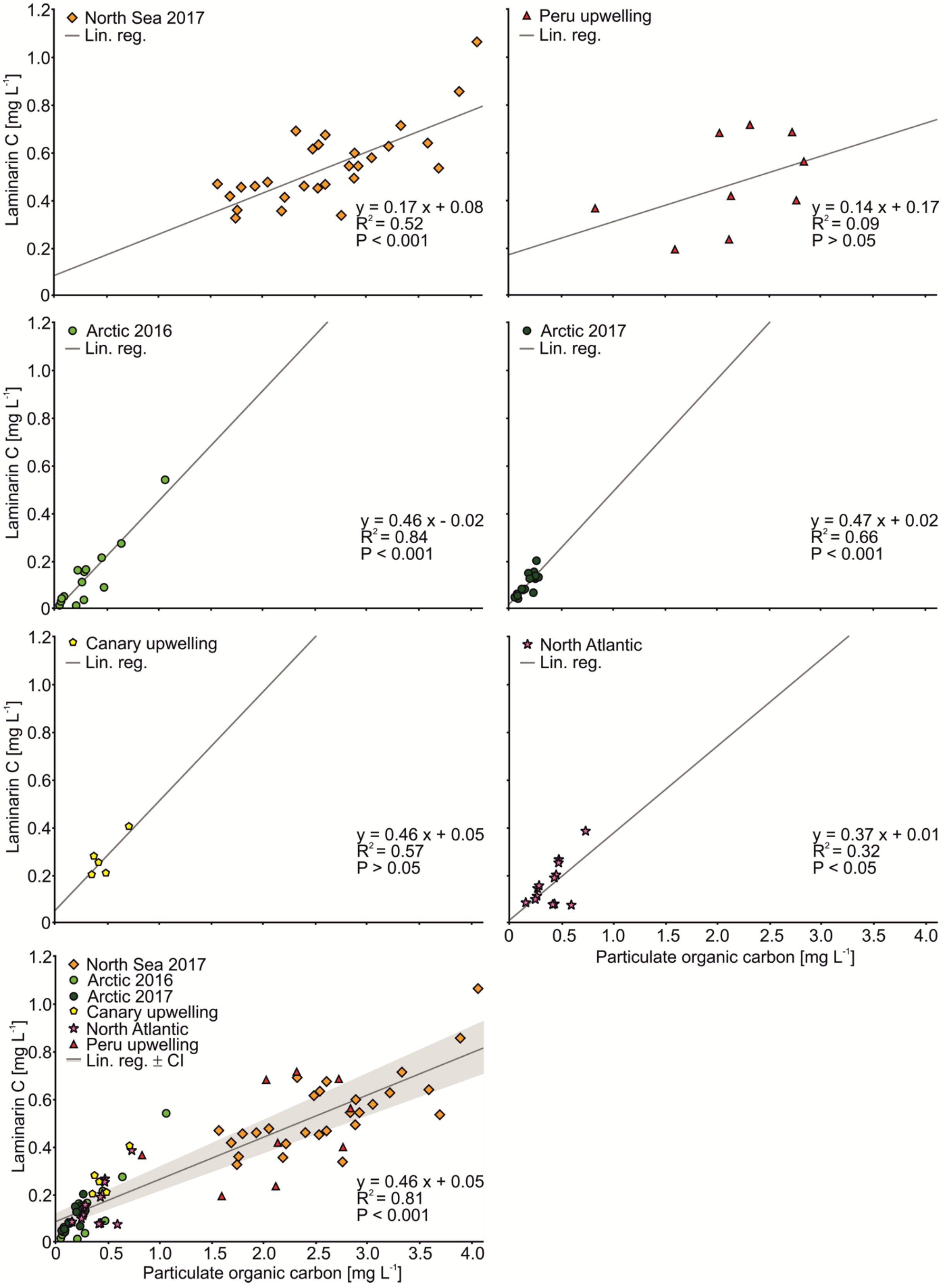

Fig. S12: Correlation of laminarin to particulate organic carbon content. The scatter plots comprise data from all regions where POC was measured. Linear regression was applied to the laminarin to POC relationship.

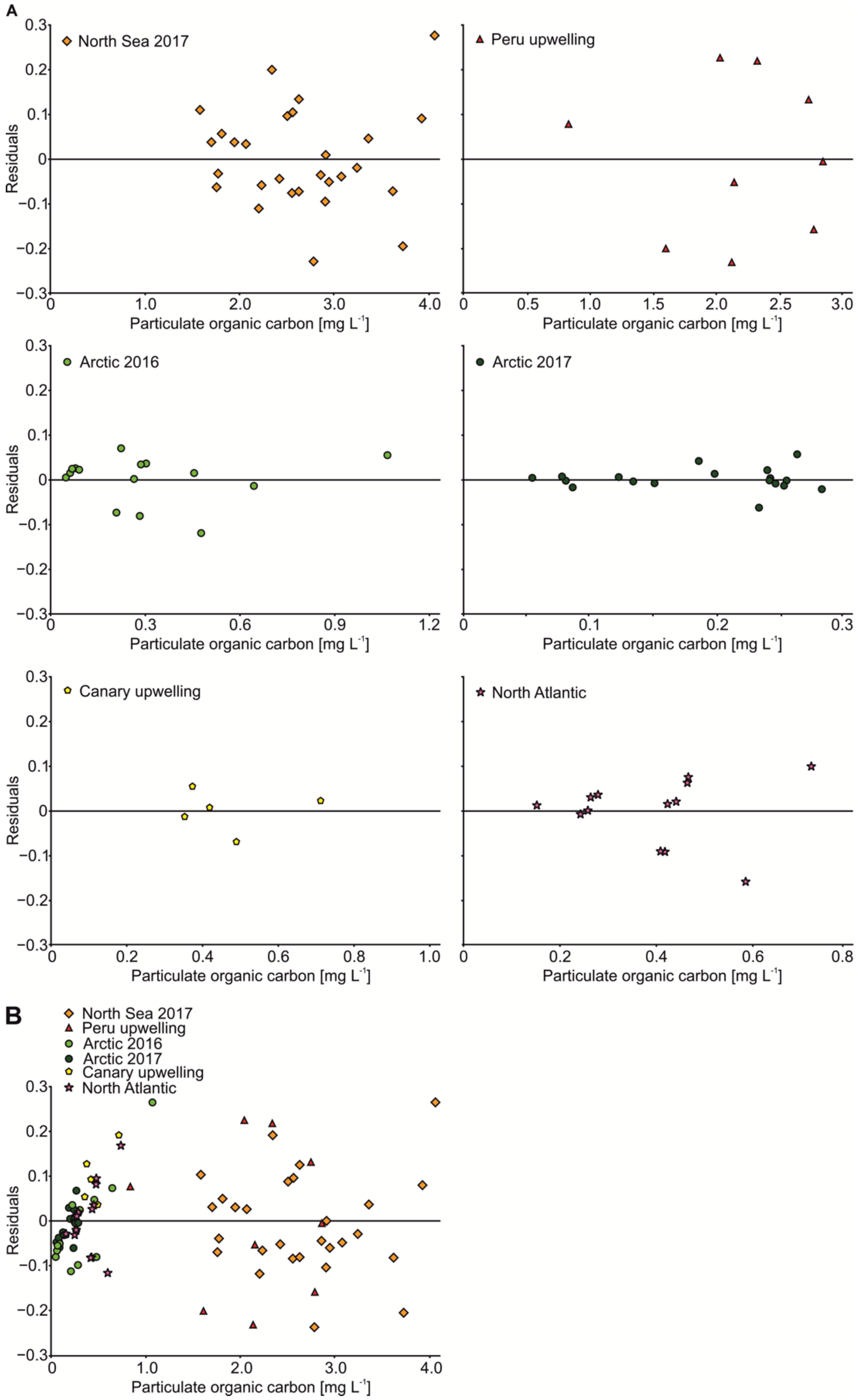

Fig. S13: Residual distributions of the linear models of laminarin C to particulate organic carbon content. (A) The scatter plot depicts the distribution from each linear regression model applied to those regions where POC was measured individually. **(B)** The integrated scatter plot comprises the overall linear model.

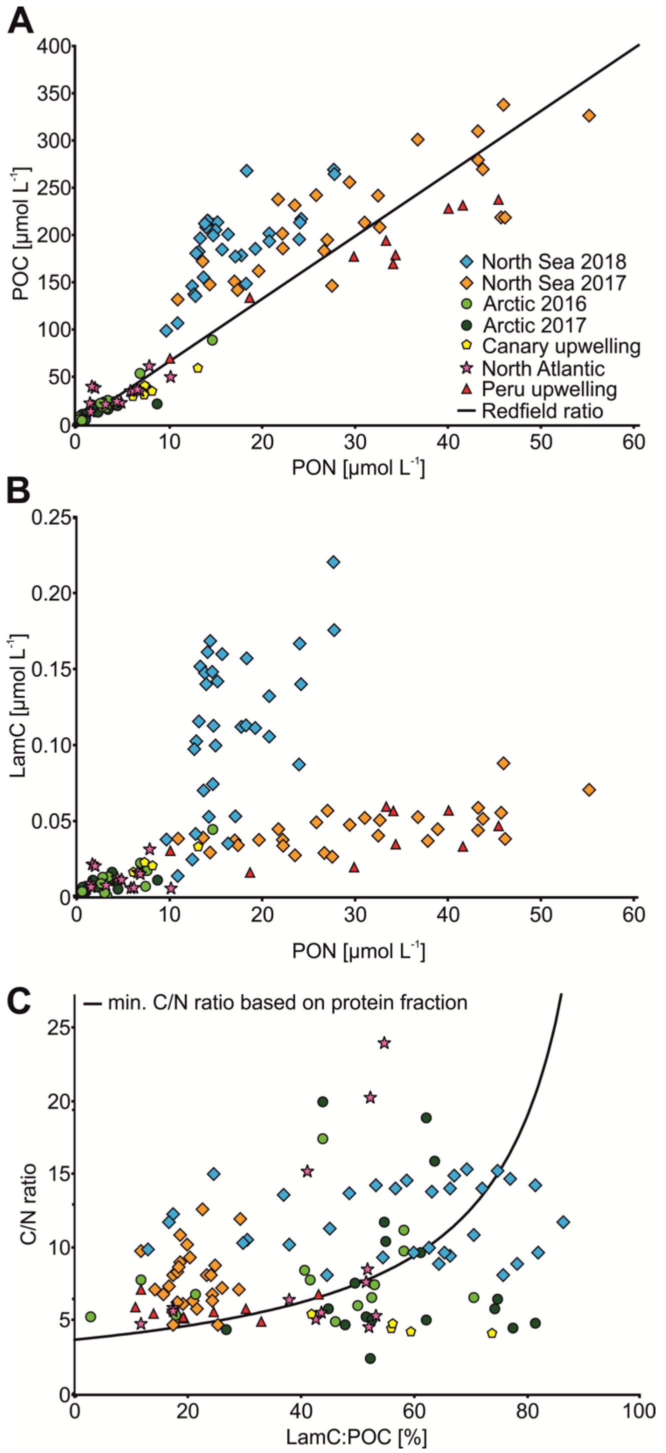

Fig. S14: Particulate organic nitrogen content (PON) compared with particulate organic carbon (POC) (A) and laminarin carbon (LamC) (B) and a confidence test of the analytical error of the two combined methods of laminarin and POC quantification (C). We tested the confidence of the LamC:POC ratios by comparing it to the theoretical minimum C/N ratio of proteins, presumably one of the largest sources of nitrogen in microalgae. Assuming that POM is a two-component mixture of the major pools glycan and protein, we can estimate a maximum permitted N content for any given laminarin concentration using the equation C/N = 3.82 * (1-f_{lam}) with 3.82 being the theoretical average

C/N value of protein and f_{lam} representing the fractional abundance of laminarin. Values below the model curve might overestimate Lam:POC ratios and were excluded from calculations to extrapolate a global laminarin production based on the measured fraction of laminarin in POC (Fig. 5).

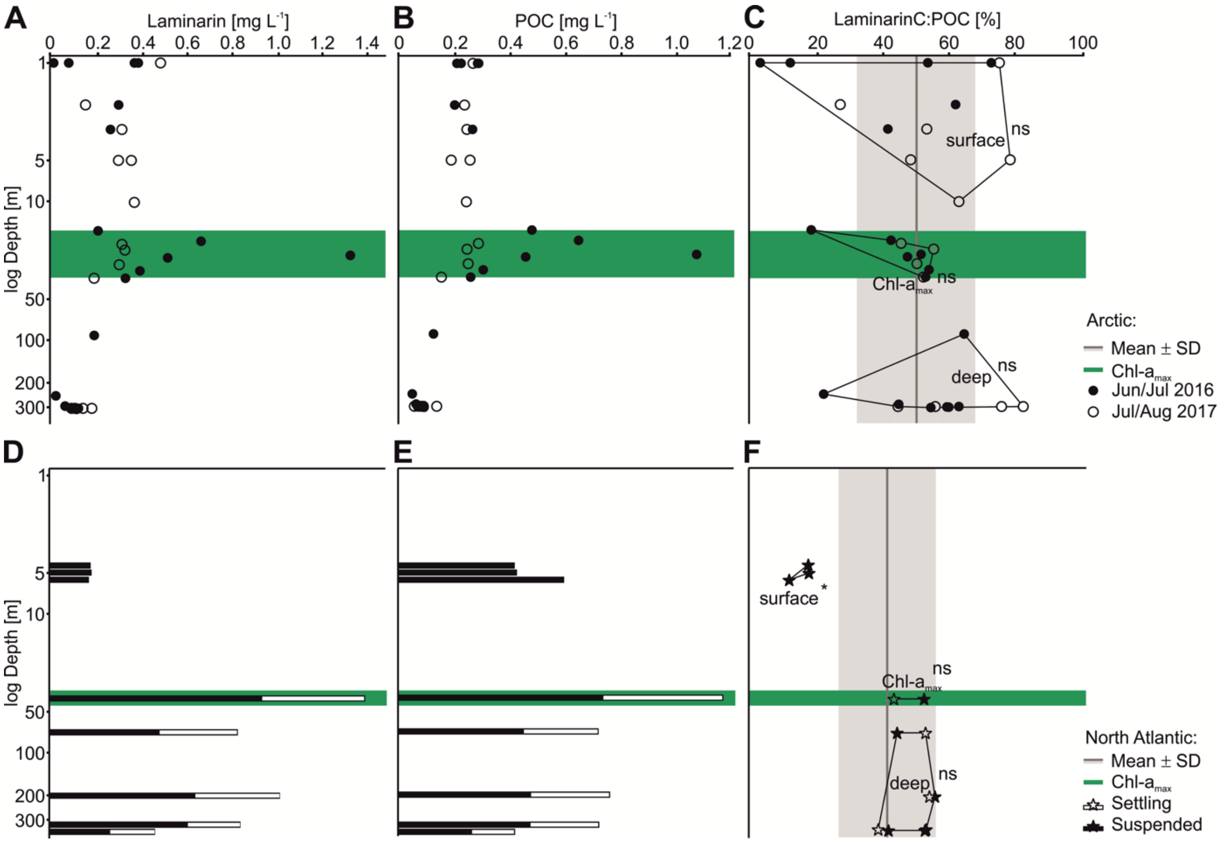

Fig. S15: Vertical profiles of laminarin, POC and laminarin:POC ratios in the Arctic and Northern Atlantic datasets. (A) Laminarin over depth during both Arctic samplings. **(B)** POC over depth during both Arctic samplings. **(C)** LaminarinC:POC ratio over depth during both Arctic samplings. Significant deviations from the overall mean LaminarinC:POC ratio of 50±18% (SD), were tested (Kruskal-Wallis-Test; ns, not significant). **(D)** Laminarin over depth during the North Atlantic sampling. **(E)** POC over depth during the North Atlantic sampling. **(F)** LaminarinC:POC ratio over depth during the North Atlantic sampling. Significant deviations from the overall mean LaminarinC:POC ratio of 41±14% (SD), were tested (Kruskal-Wallis-Test; *, $P<0.05$; ns, not significant).

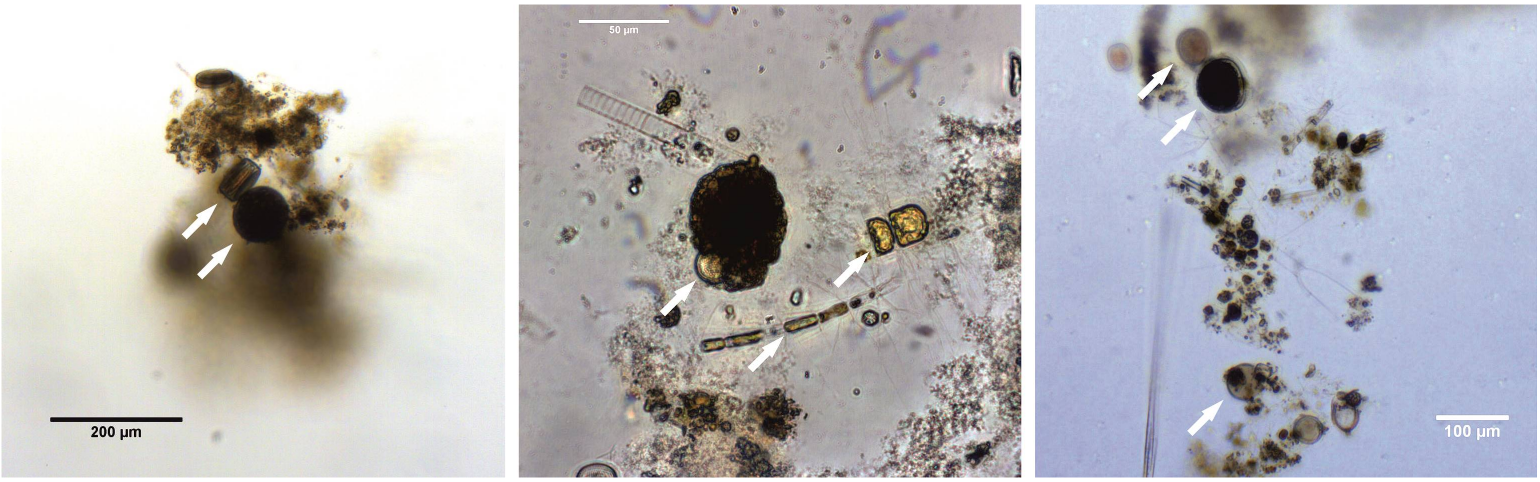

Fig. S16: Images of marine snow particles and microalgal cells. White arrows indicate structures that are characteristic for intact centric and pennate diatoms. The samples were taken by a marine snow catcher during the cruise DY077 to the North Atlantic (Porcupine Abyssal Plains). They were taken at the Chl-a maximum at 40 m water depth and resulted in two samples of the sinking particle and suspended fraction.

Tab. S1: Overview of the entire dataset, including sample types, filters used and analyses performed.

Dataset	# Depths	# Days of time series	# Stations	# Laminarin samples PC* 10/3µm	# Laminarin samples GF/D† 3µm	# Laminarin samples GF/F† 0.7µm	# POC‡ samples	# Chlorophyll a samples§ Total	# Chlorophyll a samples§ Group fractions¶	# suspended/settling particle samples‖	# Protein/lipid samples⊥
Arctic 2016	3	NA°	5	NA	15	0	15	14	NA	NA	15
Arctic 2017	3	NA	7	NA	18	0	18	18	NA	NA	NA
North Sea 2016	1	93	1	18	18	0	NA	61	NA	NA	NA
North Sea 2017	1	106	1	28	0	28	28	72	72	NA	NA
North Atlantic	4	NA	3	NA	13	0	13	NA	NA	10	NA
Mid-Atlantic	1	NA	23	23	NA	NA	NA	23	NA	NA	NA
Canary upwelling	1	NA	5	NA	5	0	5	NA	NA	NA	NA
Peru upwelling	1	NA	9	NA	9	0	9	NA	NA	NA	NA
			54	69	78	28	88	188	72	10	15

The entire dataset comprises samples from 8 separate cruises or campaigns. All samples were filtered on either polycarbonate* (PC) or glass fiber filter † (GF/D or GF/F) and the volume was logged. The North Sea surface water was sampled during two time series campaigns in spring 2016 (from March to June) and 2017 (from January to May) at the island of Helgoland in the German Bight (Fig.S2). The Arctic was sampled during the two cruises PS99.2 in 2016 and PS107 in 2017 at 8 different stations in total. Different water depths were sampled in situ by deploying several large volume water transfer systems at the same time. The Mid-Atlantic sea surface was sampled during the Atlantic Meridional Transect 22 (AMT) cruise in 2012 at 23 stations. The Canary upwelling system was sampled during the POS508 cruise in 2017 at 5 stations. The Peru upwelling system was sampled during the M138 cruise in 2017 at 9 stations. The North Atlantic was additionally sampled during the DY077 cruise in 2017 at 3 stations in the area of the Porcupine Abyssal Plains. ‖ Sea surface water was sampled by using the ship onboard flowing seawater system and additionally a marine snow catcher was used at different depths to fractionate suspended and settling particle samples. ‡ Particulate organic carbon quantification was performed by elemental analysis. § Chlorophyll a was either determined via spectrofluorometric analysis or HPLC. ¶ Multi-wavelength analysis provided an estimate for the chlorophyll contributions of certain group fractions. ⊥ Protein and lipid was determined via an adapted Lowry assay and extraction and weighing, respectively. ○ *NA* data was not determined. Sums are shown in the last row.

Tab. S2: Extracellular hydrolysis rates of laminarin in various oceanic regions.

	Extracellular laminarin hydrolysis rate [nmol monomer L^{-1} h^{-1}]	Number of stations	Region
Arnosti 2000	0.1 ± 0.002	1	North Sea
Arnosti *et al.* 2005	1.2 - 14.6	8	global
Arnosti 2008	1.4 - 2.5	1	Arctic
Ziervogel and Arnosti 2009	11.6 - 34.2	4	Delaware Estuary
Arnosti *et al.* 2009	30.2 ± 3.1	2	Gulf of Mexico
Ziervogel *et al.* 2010	~ 14 - 18	1	Gulf of Mexico
Arnosti *et al.* 2011	0.2 - 21.3	32	global
Arnosti *et al.* 2012	10.0 - 23.6	4	North Atlantic
Arnosti and Steen 2013	~ 0.5 - 20	3	Gulf of Mexico/ Arctic
Reintjes *et al.* 2018	1.6 - 22.0	5	Mid-Atlantic
Reintjes *et al.* in prep.	~ 3.9	1	North Sea
Range	~ 0.1 - 34		
